# Evolution of plant phage-type RNA polymerases: the genome of the basal angiosperm *Nuphar advena *encodes two mitochondrial and one plastid phage-type RNA polymerases

**DOI:** 10.1186/1471-2148-10-379

**Published:** 2010-12-06

**Authors:** Chang Yin, Uwe Richter, Thomas Börner, Andreas Weihe

**Affiliations:** 1 Institut für Biologie, Humboldt-Universität zu Berlin, Chausseestr. 117, 10115 Berlin, Germany; 2 Dept. of Genetics and Biotechnology, Faculty of Agricultural Sciences, Aarhus University, Forsøgsvej 1, DK-4200 Slagelse, Denmark; 3 FinMIT, Mol. Neurology, Biomedicum, University of Helsinki, Haartmaninkatu 8, 00290 Helsinki, Finland

## Abstract

**Background:**

In mono- and eudicotyledonous plants, a small nuclear gene family (*RpoT, R*NA *po*lymerase of the *T*3/*T*7 type) encodes mitochondrial as well as chloroplast RNA polymerases homologous to the T-odd bacteriophage enzymes. *RpoT *genes from angiosperms are well characterized, whereas data from deeper branching plant species are limited to the moss *Physcomitrella *and the spikemoss *Selaginella*. To further elucidate the molecular evolution of the RpoT polymerases in the plant kingdom and to get more insight into the potential importance of having more than one phage-type RNA polymerase (RNAP) available, we searched for the respective genes in the basal angiosperm *Nuphar advena*.

**Results:**

By screening a set of BAC library filters, three *RpoT *genes were identified. Both genomic gene sequences and full-length cDNAs were determined. The *NaRpoT *mRNAs specify putative polypeptides of 996, 990 and 985 amino acids, respectively. All three genes comprise 19 exons and 18 introns, conserved in their positions with those known from *RpoT *genes of other land plants. The encoded proteins show a high degree of conservation at the amino acid sequence level, including all functional crucial regions and residues known from the phage T7 RNAP. The N-terminal transit peptides of two of the encoded polymerases, NaRpoTm1 and NaRpoTm2, conferred targeting of green fluorescent protein (GFP) exclusively to mitochondria, whereas the third polymerase, NaRpoTp, was targeted to chloroplasts. Remarkably, translation of NaRpoTp mRNA has to be initiated at a CUG codon to generate a functional plastid transit peptide. Thus, besides *AGAMOUS *in *Arabidopsis *and the *Nicotiana RpoTp *gene, *N. advena RpoTp *provides another example for a plant mRNA that is exclusively translated from a non-AUG codon. In contrast to the RpoT of the lycophyte *Selaginella *and those of the moss *Physcomitrella*, which are according to phylogenetic analyses in sister positions to all other phage-type polymerases of angiosperms, the *Nuphar *RpoTs clustered with the well separated clades of mitochondrial (NaRpoTm1 and NaRpoTm2) and plastid (NaRpoTp) polymerases.

**Conclusions:**

*Nuphar advena *encodes two mitochondrial and one plastid phage-type RNAP. Identification of a plastid-localized phage-type RNAP in this basal angiosperm, orthologous to all other RpoTp enzymes of flowering plants, suggests that the duplication event giving rise to a nuclear gene-encoded plastid RNA polymerase, not present in lycopods, took place after the split of lycopods from all other tracheophytes. A dual-targeted mitochondrial and plastididal RNA polymerase (RpoTmp), as present in eudicots but not monocots, was not detected in *Nuphar *suggesting that its occurrence is an evolutionary novelty of eudicotyledonous plants like *Arabidopsis*.

## Background

In the mitochondria of all eukaryotes, with the exception of jacobids, the bacterial-type RNA polymerase of the former endosymbiont has been replaced by a T-odd phage-type RNA polymerase (for review, see [1]). The mitochondrial genome of the jacobid *Reclinomonas americana *encodes a bacterial-type RNAP [2,3], whose expression has still to be demonstrated. Likewise, chloroplast genomes have retained the *rpoA*, *B*, and *C *genes of their cyanobacterial ancestor, which encode the core subunits of the *p*lastid-*e*ncoded *p*lastid RNAP (PEP). Additionally, mono- and eudicotyledonous plants were found to require a second, *n*uclear gene-*e*ncoded *p*lastid RNAP activity (NEP) to transcribe their chloroplast genes [1,4,5]. Phage-type RNA polymerases were identified as representing this NEP activity [6-8]. Thus, in mono- and eudicots, nuclear gene-encoded phage-type RNA polymerases (RpoT polymerases) not only transcribe the mitochondrial genome but are also involved in the transcription of the plastid genome [1,5,9]. Genes encoding phage-type RNA polymerases have been identified in the nuclear genomes of various flowering plants, like *Chenopodium album *[10], *Arabidopsis thaliana *[7,11], *Nicotiana *ssp. [12-14], *Zea mays *[15], wheat [16], barley [17], and rice [18]. The moss *Physcomitrella patens *contains three *RpoT *genes [19,20], genome project data, http://www.phytozome.net/physcomitrella. Two of the *Physcomitrella *RpoTs are potentially capable of being targeted to both mitochondria and chloroplasts [19], whereas the third gene encodes an RNAP of exclusively mitochondrial localization (U. Richter, unpublished data). Eudicots like *Arabidopsis *and *Nicotiana *harbor three phage-type RNA polymerases as well, but their localization within the cell differs from the *Physcomitrella *enzymes. Eudicots possess a mitochondrial (RpoTm), a plastid (RpoTp) and a dual-targeted phage-type RNA polymerase (RpoTmp; [11,13,14]), the latter involved in the transcription of mitochondrial and plastid genes [21-24]. No phage-type NEP has been detected in algae thus far. In *Chlamydomonas*, only one *RpoT *gene was identified (Weihe et al., unpublished data; genome project data, http://genome.jgi-psf.org/Chlre4/Chlre4.home.html), presumably encoding a mitochondrial-localized RNAP. The single-copy *RpoT *genes identified in the genomes of other green algae (*Ostreococcus, Micromonas*), most likely, encode mitochondrial RNA polymerases. Multiple phage-type RNA polymerases are only found in land plant species. Maier and colleagues [25] proposed that this feature could either be a prerequisite for the spatio-temporal regulatory needs of embryophytes and an adaption to the peculiar requirements of a terrestrial life style or it might be the mere result of the specifics of the plant organelle genetic systems in interaction with the nuclear genome (transgenomic suppression of point mutations). In this context it is interesting to note that the lycophyte *Selaginella moellendorffii *possesses also only a single RpoT polymerase, which likely is exclusively active in mitochondria [26]. Thus, there seems to be no NEP activity in the lycophytes. Like the *Physcomitrella *RpoTs, the *Selaginella *polymerase is separated in phylogentic trees from the angiosperm clade, which forms two groups: plastid-localized enzymes on one hand, and mitochondrial and dual-targeted polymerases on the other [1,5]. The origin of the NEP activity as found in mono- and eudicots and of the dual-targeted RpoT polymerases observed in eudicots remains unclear.

To gain a deeper insight into the evolution of phage-type RNA polymerases in the plant lineage and to deepen our understanding of the significance of multiple phage-type RNAP activities in both mitochondria and plastids we have investigated the waterlily *Nuphar advena*. Together with *Amborella, Liriodendron *and *Acorus*, *Nuphar *is one of the most studied basal angiosperms. As one of the deepest branching angiosperms, *Nuphar *has become an important model plant for understanding the origin of key angiosperm innovations. Here, we report the identification and characterization of three *RpoT *genes from *Nuphar advena*. Our data indicate that *Nuphar advena *(and possibly other basal angiosperms) possesses two mitochondrial-localized phage-type RNAPs as well as already a plastid-localized polymerase.

## Results

### *Nuphar advena *possesses three *RpoT *genes

**Figure 1:**
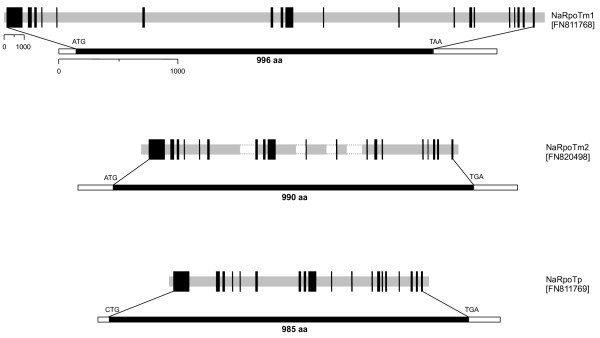
***Nuphar advena *encodes three phage-type RNA polymerases**. Schematic representation of the three *NaRpoT *genes. Coding (black) and non-coding (gray) regions are specified on the genomic sequences. Corresponding cDNA sequences, comprising the complete *RpoT *reading frames, are shown next to the genomic sequences. Positions of start (ATG, CTG, see text) and stop codons (TAA, TGA), as well as the length of derived polypeptides are indicated for cDNAs.

Screening of a BAC library identified three different *RpoT *genes in *N. advena*. 24 BAC clones hybridized with an *RpoT *cDNA fragment from *Selaginella *used as probe. PCR and sequencing suggested that they represented three similar, yet individual genes. Two of these genes have been sequenced completely, the third one in large portions, including all exons (see Figure 1). The genes were named, according to subcellular localization (see below) of their gene products, *NaRpoTm1*, *NaRpoTm2*, and *NaRpoTp*. The sequences of the three *NaRpoT *genes were deposited in the EMBL database under accession numbers FN811768 (*NaRpoTm1*), FN820498 (*NaRpoTm2*) and FN811769 (*NaRpoTp*), respectively. The lengths of the three genes were 28.5 kb for *NaRpoTm1*, > 16.2 kb for *NaRpoTm2*, and 13.6 kb for *NaRpoTp*.

### Isolation of *Nuphar RpoT *cDNAs

**Figure 2:**
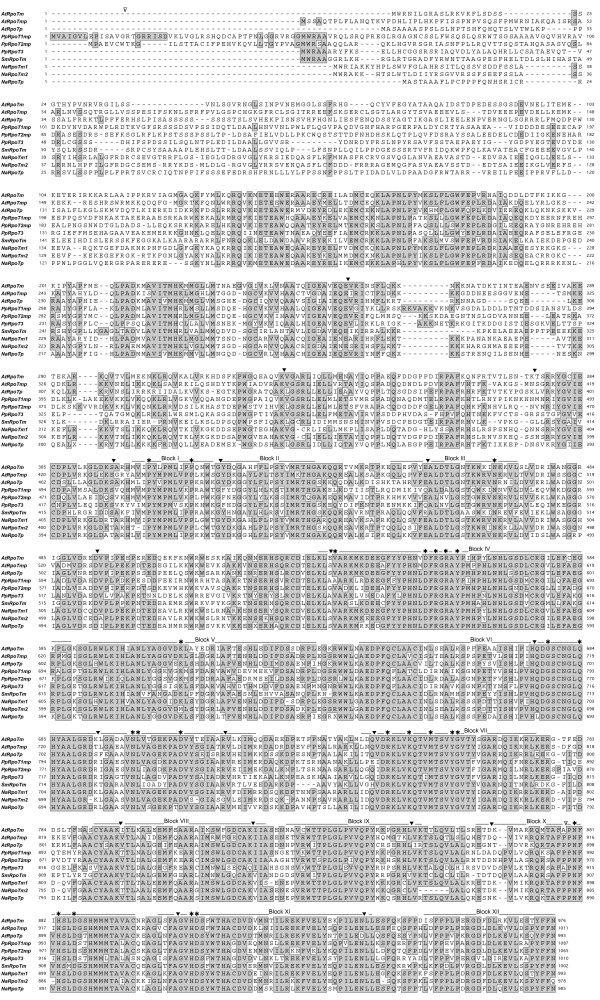
**Comparison of the deduced amino acid sequences of RpoT polymerases**. Sequences from *Nuphar *(NaRpoTm1, NaRpoTm2 and NaRpoTp), *Selaginella *(SmRpoTm), *Arabidopsis *(AtRpoTm, AtRpoTp and AtRpoTmp) and *Physcomitrella *(PpRpoT1mp, PpRpoT2mp and PpRpoT3) were aligned using ClustalW. Accession numbers are as follows: AtRpoTm, P92969; AtRpoTmp, CAC17120; AtRpoTp, O24600; PpRpoTmp1, CAC95163; and PpRpoTmp2, CAC95164. PpRpoT3 is an RpoT amino acid sequence derived from the database of the *Physcomitrella patens *genome project http://www.phytozome.net/physcomitrella. *In silico *analysis of the genome as well as expressed sequence tag (EST) data strongly suggest that the sequence, designated as PpRpoT3, is a product of an *RpoT *gene with the conserved intron-exon structure of land plants that encodes a functional RNA polymerase (U. Richter, unpublished data). Black lines indicate conserved blocks in the RpoT polymerase family; functionally crucial residues [28,29] are indicated by asterisks. The position of common introns is designated by filled triangles and PpRpoT2mp-specific introns by open triangles. Conserved amino acid positions (60%) are shaded.

Full-length cDNAs were obtained by RACE (rapid amplification of cDNA ends) reactions using specific primers (for primer sequences, see Additional file 1) derived from the genomic sequences as shown in Figure 1. All angiosperm nuclear *RpoT *genes identified thus far comprise 18 introns at conserved positions [1]. Comparison of genomic and cDNA sequences (see Figure 1) shows that these 18 introns are present as well, at the same insertion sites (see Figure 2), in the three *Nuphar RpoT *genes. None of the additional introns found in the 5' part of the *Physcomitrella *and *Selaginella RpoT *genes, respectively, were found in the *Nuphar *genes. The lengths of the introns vary considerably among the three *Nuphar RpoTs*, and most of the introns are much longer than those of other land plant *RpoT *genes. All exon-intron junctions contain conserved GT and AG sequences at the 5'- and 3'- ends of the introns, respectively.

Remarkably, *NaRpoTp *did not exhibit the canonical translation start codon ATG (AUG). Instead, a CTG (CUG) codon was found at position +148, from which translation could be initiated. The following findings are indicative of a translation start from this position: Stop codons in the 5' region exclude further upstream translation initiation sites. The methionine encoded by the most upstream in-frame ATG (nt 466 of *NaRpoTp*) aligns to amino acid residue 125 of *Arabidopsis *RpoTp, and the amino terminus derived from this position displayed neither plastid nor mitochondrial targeting properties (see below). On the other hand, the deduced amino acid sequence starting at +148 is enriched in hydroxylated amino acids, but is virtually lacking acidic residues, thus exhibiting features of stroma-targeting plastid transit peptides [27]. Interestingly, a translational start from a CUG codon has been found in the *RpoTp *gene of tobacco [12]. Thus, we assume that translation of *NaRpoTp *starts from a non-canonical CUG at position +148.

The predicted NaRpoT proteins comprise 996 (NaRpoTm1), 990 (NaRpoTm2) and 985 (NaRpoTp) amino acids, respectively. NaRpoTm1 and NaRpoTm2 exhibit a remarkably high identity of 96.8%, NaRpoTp has 63.1% and 64.6% identical residues compared with NaRpoTm1 and NaRpoTm2, respectively. The alignment of the RpoT polymerases from *N. advena *with those from *Arabidopsis*, *Physcomitrella *and *Selaginella *(see Figure 2) demonstrates a high degree of conservation at the amino acid sequence level, most striking in the C-terminal part, including all functionally crucial regions and residues known from the phage T7 RNA polymerase [28,29].

### Targeting of the *N. advena *RpoTm1 and RpoTm2 polymerases

**Figure 3:**
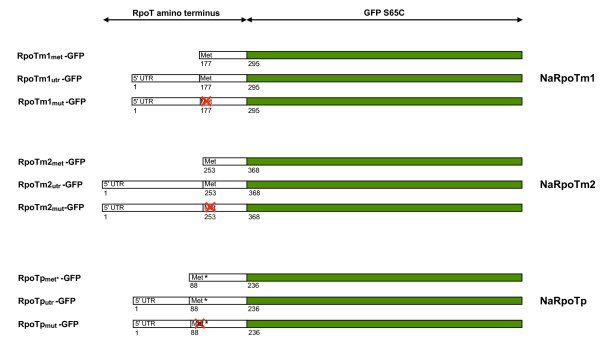
**GFP fusion constructs for targeting experiments**. Amino-terminal RpoT sequences (white bars) were translationally fused to GFP S65C (green bars) in plasmid pOL (see "Methods"). The lengths of the fragments are given by nucleotide numbers (+1 is the 5' end of the 5'-UTR). The translation start is indicated by Met or Met* (CUG-coded start codon); the crossed Met (Met*) position designates the mutation introduced at that position to prevent initiation of translation.

**Figure 4:**
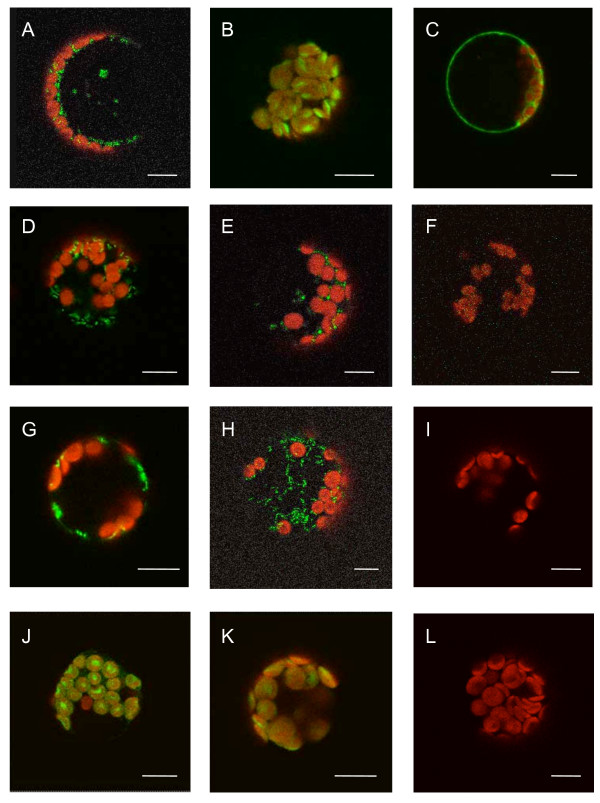
**Subcellular localization of *NaRpoT *gene products**. Confocal laser scanning microscopy of transformed Arabidopsis protoplasts. The images depict fluorescence patterns (merged green and red channels) of control constructs targeting GFP to mitochondria (**A**), plastids (**B**), vector control containing no transit peptide (**C**), Na-RpoTm1_met_-GFP (**D**), Na-RpoTm1_utr_-GFP (**E**), Na-RpoTm1_mut_-GFP (**F**), Na-RpoTm2_met_-GFP (**G**), Na-RpoTm2_utr_-GFP (**H**), Na-RpoTm2_mut_-GFP (**I**), Na-RpoTp_met_*-GFP (**J**), Na-RpoTp_utr_-GFP (**K**) and Na-RpoTp_mut_-GFP (**L**). Scale bar = 10 μm.

Subcellular localization of the *Nuphar RpoT *gene products was predicted using the algorithms TargetP [30]http://www.cbs.dtu.dk/services/TargetP and Predotar [31]http://urgi.versailles.inra.fr/predotar/predotar.html. For NaRpoTm1 and NaRpoTm2 both algorithms specified a mitochondrial import of the proteins, whereas analysis of NaRpoTp clearly indicated plastid targeting properties. To verify the subcellular localization, the amino termini of the *Nuphar *RpoT sequences were translationally fused to GFP (Figure 3). Assuming that translation starts from the first encoded methionine, the following constructs were generated: *Na-RpoTm1_met_-GFP *and *Na-RpoTm2_met_-GFP *with the first encoded methionine cloned immediately downstream of the 35 S promoter for forced translation initiation, *Na-RpoTm1_utr_-GFP *and *Na-RpoTm2_utr_-GFP *containing the whole 5' untranslated region, and *Na-RpoTm1_mut_-GFP *and *Na-RpoTm2_mut_-GFP*, in which the encoded methionine had been substituted by isoleucine (see Figure 3). The fusion proteins were expressed in *Arabidopsis *protoplasts. The results of the subcellular import studies are presented in Figure 4. Transformation with the mitochondrial control CoxIV-GFP [32] resulted in accumulation of GFP in punctuate structures of about 1 μm size (Figure 4A) identified as mitochondria [7,11]. A GFP fusion of the amino terminus of *Arabidopsis *RecA [32] was employed as a plastid control (Figure 4B). In accordance with the targeting predictions, both Na-RpoTm1-GFP and Na-RpoTm2-GFP constructs exhibited the same characteristic subcellular localization: in the case of Na-RpoTm1_met_-GFP (Figure 4D) and Na-RpoTm2_met_-GFP (Figure 4G), with forced translation from the first encoded methionine, GFP fluorescence was observed exclusively in mitochondria. The constructs containing the full-length of the 5' untranslated leader sequence, Na-RpoTm1_utr_-GFP (Figure 4E) and Na-RpoTm2_utr_-GFP (Figure 4H) showed exclusive mitochondrial targeting as well. When the mutated (preventing recognition of the AUG codon) transit peptides Na-RpoTm1_mut _(Figure 4F) and Na-RpoTm2_mut _(Figure 4I) were used, GFP fluorescence was detectable neither in mitochondria, nor in chloroplasts. It was concluded that the AUG at position +177 (NaRpoTm1) and +253 (NaRpoTm2), respectively, are the only available *RpoT *start codons, from which translation of polypeptides with mitochondrial targeting properties is initiated.

### *Nuphar *RpoTp translation is efficiently initiated at a CUG codon

Examination of *NaRpoTp *upstream sequences revealed a CTG triplet at nucleotide position +148 (see above). Translation initiation at this CUG codon would give rise to an RpoTp protein of 985 residues, the amino terminus of which was predicted *in silico *to possess plastid targeting properties. To experimentally test whether translation indeed initiates at this non-canonical codon, the following three Na-RpoTp-GFP constructs were generated (see Figure 3): Na-RpoTp_met_*-GFP, with the wild-type CUG (+148) cloned immediately downstream of the 35 S promoter for forced translation; Na-RpoTp_utr_-GFP containing the whole 5' untranslated region of 236 nt and thus preserving the sequence context, known to be crucial for initiation at non-AUG codons in plants [33]; and Na-RpoTp_mut_-GFP, in which the CUG was modified to CAC to prevent the recognition of CUG as a startcodon. The Na-RpoTp_met_*-GFP construct gave rise to green GFP fluorescence in chloroplasts which overlapped with the red chlorophyll autofluorescence, clearly confirming co-localization of red and green fluorescence in chloroplasts (Figure 4J). An identical fluorescence pattern was observed using construct Na-RpoTp_utr_-GFP (Figure 4K), whereas expression of *Na-RpoTp_mut_-GFP *(Figure 4L) completely abolished import of the GFP to the chloroplasts. These data provide convincing evidence that translation of *NaRpoTp *is solely initiated from the CUG codon at position +148.

### Phylogenetic analysis

**Figure 5:**
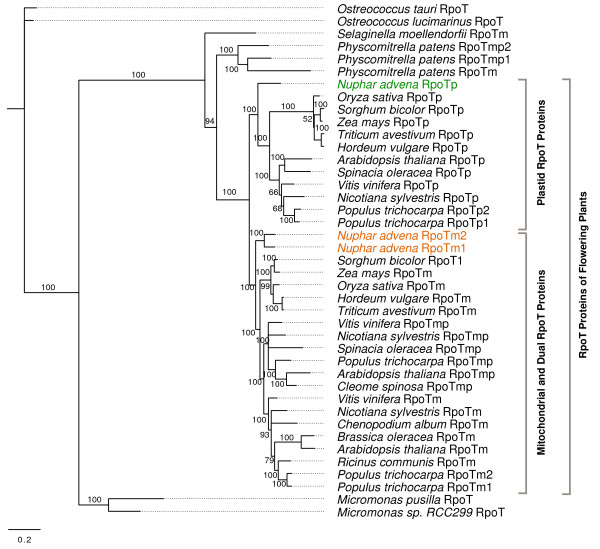
**Phylogenetic analysis of RpoT sequences**. ML (Bayesian) tree of plant RpoT protein sequences based on an alignment of conserved blocks (see "Methods"). For accession numbers of the sequences, see Additional file 2.

Using the Bayesian algorithm, maximum-likelihood (ML) as well as maximum parsimony (MP), phylogenetic trees were reconstructed to elucidate the molecular phylogeny of the RpoT polymerases and to determine the evolutionary position of the polymerases identified and described in the present study. Tree reconstruction was based on a multiple alignment of 41 RpoT sequences (see "Methods"). Bayesian as well as ML and MP analysis resulted in essentially the same topology (not shown). Figure 5 shows the consensus tree of a Bayesian analysis in which angiosperm RpoT polymerases constitute two clearly discernible groups: one consisting of plastid-localized polymerases, and the other of mitochondrial-localized and dual-targeted enzymes. Whereas the *Selaginella *and *Physcomitrella *polymerases do not belong to the branches of well separated plastid and mitochondrial (and dual targeted) polymerases, the RpoT polymerases from the basal angiosperm *N. advena *cluster with the branches of plastid and mitochondrial/dual targeted sequences: NaRpoTm1 and NaRpoTm2 within the mitochondrial, and NaRpoTp within the plastid branch.

## Discussion

Genes encoding phage-type mitochondrial and plastid RNA polymerases have been identified from numerous monocotyledonous and eudicotyledonous angiosperm species (for review, see [1]). In contrast, knowledge on RpoT polymerases of deep branching land plants is so far limited to the moss *Physcomitrella patens *[19,20] and the lycophyte *Selaginella moellendorfii *[26], and no information at all is available about phage-type RNA polymerases from the basal angiosperm lineages that precede the monocot-eudicot divergence. Here we show that the waterlily *Nuphar advena*, a basal angiosperm, encodes three RpoT polymerases. The encoded proteins of 996, 990, and 985 amino acids, respectively, exhibit the characteristic domains that are highly conserved between all RpoT polymerases, including the residues shown to be essential and located within the catalytic pocket of the polymerase (D537, K631, Y639, G640, D812, residue numbers as given for T7 RNA polymerase). The high conservation of amino acid sequences and the identical position of the introns in the *RpoT *genes of *Selaginella*, *Physcomitrella, Nuphar *and monocotyledonous and eudicotyledonous angiosperms (see Figure 2) suggests a common ancestral gene giving rise to all land plant *RpoT *genes. Phylogenetic analysis (see Figure 5) confirms this hypothesis.

Although *Physcomitrella *(one mitochondrial and two dual-targeted) and eudictos (one mitochondrial, one plastid and one dual-targeted) possess also three phage-type RNA polymerases, the localization of the three *Nuphar *RpoT polymerases shows a new pattern. The N-termini of two of the three *RpoT *genes of *N. advena *show properties of mitochondrial transit peptides. Using translational fusions of the putative NaRpoT transit peptides with GFP, we demonstrated that these transit peptides confer exclusively mitochondrial import. Mitochondrial import of NaRpoTm1- and NaRpoTm2-GFP was also maintained when the fusion constructs contained the full-length 5'-UTRs of the genes (Figure 4). We included these constructs in our study since the presence of the 5'-UTR may alter the targeting of proteins [34]. Thus, we conclude that *N. advena *encodes two phage-type mitochondrial RNA polymerases. Phylogenetic analysis (see Figure 5) indicates that the third *RpoT *gene of *Nuphar*, *NaRpoTp*, encodes a plastid phage-type RNA polymerase. In the 5' part of the *NaRpoTp *cDNA no canonical start codon was identified, with the first ATG triplet occurring only at position 466. However, a potential non-AUG initiation codon (CUG) was revealed at position 148. Translation from this codon would yield an N-terminal leader peptide with genuine plastid targeting properties, as predicted by two prediction algorithms (TargetP and Predotar). Three different GFP fusions were designed to test the translation initiation capacity of this CUG codon. The results proved a plastid import of the derived amino-terminus (Figure 4J), as well as an efficient translation initiation at the CUG within the context of the full-length 5'-UTR (Figure 4K) that could be abolished by modifying the codon to CAC (Figure 4L). Thus, *Nuphar RpoTp *belongs to the rare cases of non-viral plant genes [35-37] that initiate translation exclusively at a non-AUG codon. Interestingly, this is the second case of non-AUG translation initiation among *RpoT *genes specifying plastid-localized RNA polymerases: translation of the tobacco *RpoTp *gene also starts from a CUG codon [12].

Both mono- and eudicotyledonous plants possess a solely plastid-localized phage-type RNAP (RpoTp) together with a purely mitochondrial-localized RpoT enzyme (RpoTm) and, in the case of eudicots, a third phage-type RNAP with dual localization in both organelles is found. The data presented here suggest that all RpoTp proteins descent from a common duplication event that took place in a common ancestor of all flowering plants. Thus far it is unknown whether ferns or gymnosperms contain nuclear genes encoding plastid-localized phage-type RNAPs as well. Since the duplication event giving rise to the second NEP activity in eudicots is clearly more recent, identification of a purely plastid-localized phage-type RNAP in the basal angiosperm *Nuphar advena*, orthologous to all other purely plastid-targeted enzymes (RpoTp) of flowering plants, suggests that the acquisition of a nuclear gene-encoded transcriptional activity for plastids, not present in lycopods, took place after the split of lycopods from all other tracheophytes, with or before the rise of flowering plants. Moreover, the lack of a dual-targeted RpoTmp both in *Nuphar *and in monocots suggests that the RpoTmp enzyme detected in eudicots is an 'invention' due to an *RpoTm *gene duplication that might have occurred only after the separation of monocots and eudicots. The putative plastid targeting sequences as present in two of the three *Physcomitrella *RpoT proteins are therefore clearly species- or lineage-specific convergent inventions. Interestingly, multiple mitochondrial RNA polymerasesas as found in *Physcomitrella *and eudicots are indentified in *Nuphar *as well. The fixation of duplicated *RpoT *genes leads to convergent multiplicity of mitochondrial RNAPs in *Nuphar*, *Physcomitrella *and eudicots, not found in any other eukaryotic lineage. Recently it was shown that in *Arabidopsis *RpoTmp null mutants transcription of a specific set of mitochondrial genes is strongly reduced. Moreover, accumulation of respiratory complexes was affected to very different levels, suggesting that the presence of multiple transcriptional activities in mitochondria may allow plants to regulate mitochondrial gene expression in a complex specific manner [24]. Further investigations will be necessary to show if a similar division of labor evolved in case of the two mitochondrial RNA polymerases in *Nuphar *and address the specific impact of NEP and PEP transcriptional activities for gene expression in *Nuphar *chloroplasts.

## Conclusions

Identification of three *RpoT *genes in *Nuphar advena*, specifying two mitochondrial and one plastid-localized polymerases, suggests that multiple phage-type organellar RNAPs already exist among basal angiosperms. From the high similarity of the encoded amino acid sequences, the conservation of intron positions and phylogenetic analysis we conclude that the *RpoT *genes of *Nuphar*, like those of *Selaginella*, *Physcomitrella *and monocotyledonous and eudicotyledonous angiosperms, trace back to a common ancestral gene giving rise to all land plant *RpoT *genes. The presence of a plastid-localized phage-type RNAP in this basal angiosperm, orthologous to all other RpoTp enzymes of flowering plants, suggests that the duplication event giving rise to a nuclear gene-encoded plastid RNA polymerase, not present in lycopods, took place after the split of lycopods from all other tracheophytes. A dual-targeted mitochondrial and plastid RNA polymerase (RpoTmp), as present in eudicots but not monocots, was not detected in *Nuphar *suggesting that this additional NEP activity (RpoTmp) is an evolutionary novelty of eudicotyledonous plants like *Arabidopsis*. Our results support the idea that *RpoT *gene duplications occurred independently of each other several times during the evolution of plants and led to different subcellular localization patterns of of organellar RNA polymerases. These data substantially extend our knowledge about the evolution of the transcriptional machineries in plant organelles.

## Methods

### Plant material and growth conditions

*Nuphar advena *were purchased from a commercial supplier (Seerosen Shop, Eschede, Germany). The plants were grown in a growth chamber at 23°C with a light/dark regime of 8/16 hr. The intensity of light in all experiments was 210 μmol photons s^-1^m^-2^.

### DNA and RNA isolation

Leaves of *N. advena *were ground to fine powder under liquid nitrogen and incubated in three volumes of CTAB buffer (2% CTAB, 1.4 M NaCl, 20 mM EDTA, 100 mM Tris-HCl, pH 8.0, 2% β-mercaptoethanol) for 1 hour with agitation at 60°C. The lysate was extracted two times with chloroform-isoamyl alcohol (24:1), and the nucleic acids were precipitated with ethanol. The DNA pellet was washed with 70% ethanol and dissolved in TE buffer (10 mM Tris-HCl, 1 mM EDTA). RNA was extracted and purified using the Concert Plant RNA Reagent (Invitrogen, Karlsruhe, Germany) and RNA Cleanup Kit (Qiagen, Hilden, Germany) according to the manufacturers' instructions.

### Isolation of cDNA and genomic cloning

cDNA cloning, screening of an *N. advena *BAC library (Nuphar_HindIII BAC; Arizona Genomics Institute, Tucson, AZ) and subcloning were performed according to standard methods [38]. A 1.5 kb cDNA fragment amplified from the 3' part of *Selaginella RpoT *[26] was used as a ^32^P-labelled hybridization probe to screen the *Nuphar *BAC library, containing 165,888 independent clones on nine individual filters, under non-stringent conditions (58°C). Identified positive clones were purchased from the Arizona Genomics Institute. BAC DNA was isolated using the QIAGEN plasmid midi kit according to the protocol of the manufacturer. Sanger dideoxy sequencing of subclones, or directly of the BAC DNA by primer walking, was performed on an ABI3130xl sequencer (Applied Biosystems, Darmstadt, Germany). From the genomic sequences obtained, primers were designed (for a list of all primers used in the present study, see Additional file 1) for rapid amplification of cDNA ends (RACE). 3'- and 5'- RACE reactions were performed with the RACE primers listed in Additional File 1 using the CapFishing kit (Seegene, Rockville, USA) and Phusion hot start DNA polymerase (Finnzyme, Espoo, Finnland) following the protocols of the manufacturers.

### Generation of targeting constructs and transient expression

The amino-terminal sequences were amplified from cDNA of the three *N. advena RpoT *genes using the primers listed in Additional file 1. Products were ligated into vector pDRIVE (Qiagen) and excised using *Xba*I and *Sal*I. The fragments were inserted into pOL-GFP [39] opened with *Spe*I and *Sal*I, to give the constructs shown in Figure 3. coxIV- and recA-GFP constructs were employed as mitochondrial and plastid control constructs [12].

All constructs were used to transfect Arabidopsis protoplasts, isolated from 3 - 5 weeks old Arabidopsis leaves grown under long day conditions (23°C, 16/8 hr light/dark), essentially as described [40]. Cell density was adjusted to 2 × 10^6^/ml. 100 μl protoplasts were transfected with 20 μg plasmid DNA in 40% polyethylene glycol 4000, 0.8 M mannitol, 1 mM CaCl_2_. Transformed protoplasts were examined two days after transfection by confocal laser scanning microscopy with a Leica TCS SP2 using 488 nm excitation and two-channel measurement of emission from 510 to 580 nm (green/GFP) and > 590 nm (red/chlorophyll).

### Phylogenetic analysis

Deduced protein sequences were aligned using ClustalW [41]. Conserved blocks were cut out and merged as described earlier [19] (see Additional file 2) and subjected to Bayesian, maximum-likelihood and maximum parsimony analysis as implemented in the Geneious program package [42,43]. The Bayesian inference method employed the Mixed amino acid replacement model with a gamma distribution to represent among-site rate heterogeneity (mixed +γ). MCMC was performed with 1 million generations and four independent chains and two runs. The Markov chain was sampled every 100 generations. Convergence was observed by plots of maximum likelihood (ML) scores and by using the run statistics. The first 20% of all trees generated were discarded; the remaining trees were used to construct a consensus tree and to calculate the posterior branch support values. In addition, maximum likelihood analysis with 1000 and maximum parsimony analysis with 1000 bootstrap replicates were conducted.

## Authors' contributions

AW and TB designed the research and outlined the manuscript. CY performed the experimental research. UR participated in the experimental work and performed computational phylogenetic analyses. CY, UR, AW and TB interpreted the data. AW and TB wrote the paper. All authors have read and approved the final manuscript.

## Supplementary Material

Additional file 1 **Oligonucleotide primers used in the experiments**. Click here for file

Additional file 2 **Merged conserved blocks of 41 RpoT sequences used for reconstruction of phylogeny**. Click here for file

## References

[B1] Weihe A Daniell H, Chase CD The transcription of plant organelle genomes Molecular biology and biotechnology of plant organelles 2004 Berlin Heidelberg New York, Springer 213 237 10.1007/978-1-4020-3166-3_8

[B2] Lang BF Burger G O'Kelly CJ Cedergren R Golding GB Lemieux C Sankoff D Turmel M Gray MW An ancestral mitochondrial DNA resembling a eubacterial genome in miniature Nature 1997 387 493 497 10.1038/387493a0 9168110

[B3] Gray MW Burger G Lang BF The origin and early evolution of mitochondria Genome Biol 2001 2 1018.1 1018.5 10.1186/gb-2001-2-6-reviews1018 PMC138944 11423013

[B4] Hess WR Börner T Organellar RNA polymerases of higher plants Int Rev Cytol 1999 190 1 59 10.1016/S0074-7696(08)62145-2 10331238

[B5] Liere K Börner T Grasser KD Transcription of plastid genes Regulation of Transcription in Plants 2007 Oxford, Blackwell Publishing 184 224 full_text

[B6] Lerbs-Mache S The 110-kDa polypeptide of spinach plastid DNA-dependent RNA polymerase: single-subunit enzyme or catalytic core of multimeric enzyme complexes? Proc Natl Acad Sci USA 1993 90 5509 5513 10.1073/pnas.90.12.5509 8516293 PMC46750

[B7] Hedtke B Börner T Weihe A Mitochondrial and chloroplast phage-type RNA polymerases in Arabidopsis Science 1997 277 809 811 10.1126/science.277.5327.809 9242608

[B8] Liere K Kaden D Maliga P Börner T Overexpression of phage-type RNA polymerase RpoTp in tobacco demonstrates its role in chloroplast transcription by recognizing a distinct promoter type Nucleic Acids Res 2004 32 1159 1165 10.1093/nar/gkh285 14973224 PMC373414

[B9] Shiina T Tsunoyama Y Nakahira Y Khan MS Plastid RNA polymerases, promoters, and transcription regulators in higher plants Int Rev Cytol 2005 244 1 68 10.1016/S0074-7696(05)44001-2 16157177

[B10] Weihe A Hedtke B Börner T Cloning and characterization of a cDNA encoding a bacteriophage-type RNA polymerase from the higher plant *Chenopodium album* Nucl Acids Res 1997 25 2319 2325 10.1093/nar/25.12.2319 9171081 PMC146756

[B11] Hedtke B Börner T Weihe A One RNA polymerase serving two genomes EMBO Rep 2000 1 435 440 10.1093/embo-reports/kvd086 11258484 PMC1083759

[B12] Hedtke B Legen J Weihe A Herrmann RG Börner T Six active phage-type RNA polymerase genes in Nicotiana tabacum Plant J 2002 30 625 637 10.1046/j.1365-313X.2002.01318.x 12061895

[B13] Kobayashi Y Dokiya Y Sugiura M Niwa Y Sugita M Genomic organization and organ-specific expression of a nuclear gene encoding phage-type RNA polymerase in Nicotiana sylvestris Gene 2001 279 33 40 10.1016/S0378-1119(01)00729-6 11722843

[B14] Kobayashi Y Dokiya Y Kumazawa Y Sugita M Non-AUG translation initiation of mRNA encoding plastid-targeted phage-type RNA polymerase in Nicotiana sylvestris Biochem Biophys Res Commun 2002 299 57 61 10.1016/S0006-291X(02)02579-2 12435389

[B15] Chang CC Sheen J Bligny M Niwa Y Lerbs-Mache S Stern DB Functional analysis of two maize cDNAs encoding T7-like RNA polymerases Plant Cell 1999 11 911 926 10.1105/tpc.11.5.911 10330475 PMC144232

[B16] Ikeda TM Gray MW Identification and characterization of T3/T7 bacteriophage-like RNA polymerase sequences in wheat Plant Mol Biol 1999 40 567 578 10.1023/A:1006203928189 10480381

[B17] Emanuel C Weihe A Graner A Hess WR Börner T Chloroplast development affects expression of phage-type RNA polymerases in barley leaves Plant J 2004 38 460 472 10.1111/j.0960-7412.2004.02060.x 15086795

[B18] Kusumi K Yara A Mitsui N Tozawa Y Iba K Characterization of a rice nuclear-encoded plastid RNA polymerase gene OsRpoTp Plant Cell Physiol 2004 45 1194 1201 10.1093/pcp/pch133 15509842

[B19] Richter U Kiessling J Hedtke B Decker E Reski R Börner T Weihe A Two *RpoT *genes of Physcomitrella patens encode phage-type RNA polymerases with dual targeting to mitochondria and plastids Gene 2002 290 95 105 10.1016/S0378-1119(02)00583-8 12062804

[B20] Kabeya Y Hashimoto K Sato N Identification and characterization of two phage-type RNA polymerase cDNAs in the moss Physcomitrella patens: implication of recent evolution of nuclear-encoded RNA polymerase of plastids in plants Plant Cell Physiol 2002 43 245 255 10.1093/pcp/pcf041 11917078

[B21] Baba K Schmidt J Espinosa-Ruiz A Villarejo A Shiina T Gardestrom P Sane AP Bhalerao RP Organellar gene transcription and early seedling development are affected in the rpoT;2 mutant of Arabidopsis Plant J 2004 38 38 48 10.1111/j.1365-313X.2004.02022.x 15053758

[B22] Courtois F Merendino L Demarsy E Mache R Lerbs-Mache S Phage-type RNA polymerase RPOTmp transcribes the rrn operon from the PC promoter at early developmental stages in Arabidopsis Plant Physiol 2007 145 712 721 10.1104/pp.107.103846 17885088 PMC2048797

[B23] Swiatecka-Hagenbruch M Emanuel C Hedtke B Liere K Börner T Impaired function of the phage-type RNA polymerase RpoTp in transcription of chloroplast genes is compensated by a second phage-type RNA polymerase Nucleic Acids Res 2008 36 785 792 10.1093/nar/gkm1111 18084023 PMC2241911

[B24] Kühn K Richter U Meyer EH Delannoy E de Longevialle AF O'Toole N Börner T Millar AH Small ID Whelan J Phage-type RNA polymerase RPOTmp performs gene-specific transcription in mitochondria of Arabidopsis thaliana Plant Cell 2009 21 2762 2779 19783760 10.1105/tpc.109.068536 PMC2768943

[B25] Maier UG Bozarth A Funk HT Zauner S Rensing SA Schmitz-Linneweber C Börner T Tillich M Complex chloroplast RNA metabolism: just debugging the genetic programme? BMC Biol 2008 6 36 10.1186/1741-7007-6-36 18755031 PMC2553071

[B26] Yin C Richter U Börner T Weihe A Evolution of phage-type RNA polymerases in higher plants: characterization of the single phage-type RNA polymerase gene from Selaginella moellendorffii J Mol Evol 2009 68 528 538 10.1007/s00239-009-9229-2 19407923

[B27] von Heijne G Steppuhn J Herrmann RG Domain structure of mitochondrial and chloroplast targeting peptides Eur J Biochem 1989 180 535 545 10.1111/j.1432-1033.1989.tb14679.x 2653818

[B28] McAllister WT Raskin CA The phage RNA polymerases are related to DNA polymerases and reverse transcriptases Mol Microbiol 1993 10 1 6 10.1111/j.1365-2958.1993.tb00897.x 7526118

[B29] Sousa R Chung YJ Rose JP Wang BC Crystal structure of bacteriophage T7 RNA polymerase at 3.3 A^o ^resolution Nature 1993 364 593 599 10.1038/364593a0 7688864

[B30] Emanuelsson O Nielsen H Brunak S von Heijne G Predicting subcellular localization of proteins based on their N-terminal amino acid sequence J Mol Biol 2000 300 1005 1016 10.1006/jmbi.2000.3903 10891285

[B31] Small I Peeters N Legeai F Lurin C Predotar: A tool for rapidly screening proteomes for N-terminal targeting sequences Proteomics 2004 4 1581 1590 10.1002/pmic.200300776 15174128

[B32] Akashi K Grandjean O Small I Potential dual targeting of an Arabidopsis archaebacterial-like histidyl-tRNA synthetase to mitochondria and chloroplasts FEBS Lett 1998 431 39 44 10.1016/S0014-5793(98)00717-0 9684861

[B33] Gordon K Fütterer J Hohn T Efficient initiation of translation at non-AUG triplets in plant cells Plant J 1992 2 809 813 10.1046/j.1365-313X.1992.t01-17-00999.x 1302633

[B34] Kabeya Y Sato N Unique translation initiation at the second AUG codon determines mitochondrial localization of the phage-type RNA polymerases in the moss Physcomitrella patens Plant Physiol 2005 138 369 382 10.1104/pp.105.059501 15834007 PMC1104190

[B35] Riechmann JL Ito T Meyerowitz EM Non-AUG initiation of AGAMOUS mRNA translation in Arabidopsis thaliana Mol Cell Biol 1999 19 8505 8512 10567575 10.1128/mcb.19.12.8505 PMC84964

[B36] Depeiges A Degroote F Espagnol MC Picard G Translation initiation by non-AUG codons in Arabidopsis thaliana transgenic plants Plant Cell Rep 2006 25 55 61 10.1007/s00299-005-0034-0 16184386

[B37] Medveczky P Nemeth A Graf L Szilagyi L Methionine-independent translation initiation from naturally occuring non-AUG codon Curr Chem Biol 2007 1 129 139 10.2174/187231307780636459

[B38] Sambrook J Fitsch EF Maniatis T Molecular Cloning: A Laboratory Manual 1989 Cold Spring Harbor, Cold Spring Harbor Press

[B39] Peeters NM Chapron A Giritch A Grandjean O Lancelin D Lhomme T Vivrel A Small I Duplication and quadruplication of Arabidopsis thaliana cysteinyl- and asparaginyl-tRNA synthetase genes of organellar origin J Mol Evol 2000 50 413 423 10824085 10.1007/s002390010044

[B40] Yo S-D Cho Y-H Sheen J Arabidopsis mesophyll protoplasts: a versatile cell system for trnsient gene expression analysis Nature Protocols 2007 2 1565 1572 10.1038/nprot.2007.199 17585298

[B41] Thompson JD Higgins DG Gibson TJ CLUSTAL W: improving the sensitivity of progressive multiple sequence alignment through sequence weighting, position-specific gap penalties and weight matrix choice Nucleic Acids Res 1994 22 4673 4680 10.1093/nar/22.22.4673 7984417 PMC308517

[B42] Drummond A Ashton B Cheung M Heled J Kearse M Moir R Stones-Havas S Thierer T Wilson A Geneious v4.0 2008 http://www.geneious.com

[B43] Guindon S Gascuel O A simple, fast, and accurate algorithm to estimate large phylogenies by maximum likelihood Syst Biol 2003 52 696 704 10.1080/10635150390235520 14530136

