# Supplementary material for: Evolution of plant phage-type RNA polymerases: the genome of the basal angiosperm Nuphar advena encodes two mitochondrial and one plastid phage-type RNA polymerases
Source: BMC Evol Biol. 2010 Dec 6;10:379. doi: 10.1186/1471-2148-10-379 (PMC3022604; doi:10.1186/1471-2148-10-379)
Supplement: Additional file 1 — Oligonucleotide primers used in the experiments. [file 1471-2148-10-379-S1.DOC]

**Additional File 1 - Oligonucleotide primers used in the experiments**

| Primers | Sequences (5’-3’) |
| --- | --- |
| *cDNA synthesis primers* |  |
| A-fw1 | CCT TGG ATA CAC TTG GGA AT |
| A-rev1 | CCG CAC CTA ACC ACC AC |
| A-fw5 | AAG GTG GTG GTT AGG TGC GGA A |
| A-rev5 | TTC CGC ACC TAA CCA CCA CCT T |
| Na-A-gap1p | TGC AAG CAG AAA CTG GCA CC |
| Na-A-gap1m | ATC AGT GAC CAT CTG CCG TCT G |
| Na-A-gap2p | GGG CTA GTG GAG GTC ACC TTG |
| Na-A-gap2m | TCC CAG GGC TGC ATA GTG TT |
| NaAgap3p1 | TAC AAA TAA CCC AAG TGC TG |
| NaAgap3m1 | CTT ATA CTG TGA CGG CAG AA |
| NaAgap3p2 | AAA ACA ACC TTG ACT GCC TT |
| NaAgap3m2 | CAG TAT CGG CTG CTC ATA GA |
| NaA-sp2p | GGG GAT TGT GCA AAG GTG ATT |
| NaA-sp2m | TAC TGT GAC GGC AGA AAT AGA A |
| C-fw1 | ACT TCT TGC GGT GGT AGA GAG A |
| C-rev1 | TCT CTC TAC CAC CGC AAG AAG T |
| C-fw2 | GGT GTG ACG TGG AAC TTA AAC TAT |
| C-rev2 | ATA GTT TAA GTT CCA CGT CAC ACC |
| C-fw3 | CTG GCA AAT CTA TAT GCT GGC GGT G |
| C-rev3 | TGC TAA ACG GCC ATC ATA |
| NaC-sp1p | GGC TGC CCT GGA GGA GAT G |
| NaC-sp1m | CTG TAA CAA AGG CTG GCT GGC T |
| NaP1p | CCC GTG CCC ACC ACC CTT C |
| NaP1m | GTG CCC ACC CTT GTC ATA CCC T |
| NaP2p | TTC CGG GAG GTC TCG TG |
| NaP2m | CAA TAG GTC TTT CTG CCG AAT C |
| NaP3p | ATG AAT GGG CAA GAC GAT GGG T |
| NaP3m1 | CAC AAT GGC TTT AAT GCG GAT G |
| NaP3m2 | TGC ACG AGC CGT CCA GGT GAA |
| NaP4p | TGA TTC GGC AGA AAG ACC TAT |
| NaP4m | TTA ATG CGG ATG CTA CAG ACA T |
| NaP10p | AAG CGT GCT ATC TTC TCC TCC |
| NaP10m | CGC CTC CGT CTC CAT CTT C |
| NaP11p | AAG TTG GGA AGC CGT CTC AT |
| NaP11m | TAG GTC TTT CTG CCG AAT CA |
| NaP12p | CAG TAA GAT GGA CCA CTC CT |
| NaP12m | CGG ATG CTA CAG ACA TGA T |
| NaP-sp1p | GTT ACA TTG TCT GCG TTG GAA G |
| NaP-sp1m | GCC ACC TTG AAA GTA ATC CC |
| *RACE primers* |  |
| NaA-rev11 | TCT TCT ATA CAA TCC GAC GCC TCT |
| NaA-rev12 | AGC CCT TGA ATT TCT TCT ATC GC |
| NaA-rev13 | AAA TCA CCA AAA TTC GGC TTG T |
| NaA-rev14 | GCC AGT TTC TGC TTG CAC ATA TC |
| NaA-fw12 | TGG GCT AGT GGA GGT CAC CTT |
| NaA-fw14 | TTT TCG TGG ACG GGC ATA TC |
| NaA-fw15 | GTT CAA GGT GGT GGT TAG GTG C |
| NaC-rev16 | AGG GTA AAT TTG GTG CCA ACT T |
| NaC-rev17 | GCC GAA GTA TCC TCA TCC GTC |
| NaC-rev18 | TCA GAA CAC CCA TCC CTA AAC C |
| NaC-fw4 | GAG AGA ATA TGG GCC AGT GGG |
| NaC-fw5 | CCT TTA CCA GAG AAG CCA GAC A |
| NaC-fw6 | GAT TTT CGA GGA CGG GCA TA |
| NaC-fw7 | AAT CTA TAT GCT GGC GGT GTT G |
| NaP-5R2 | GCC CAT TCA TCA ACA GCA CCA T |
| NaP-5R4 | CGC TCC TGC ACG AGA CCT CC |
| NaP-5R5 | AGC CAA GGT GGG TCG AGG AG |
| NaP-5R6 | GGA GGG AAA TTG GAA GAA GGA A |
| NaP-5R8 | CCT GGA GTG ATT CTG GAA GGG |
| NaP-3R1 | CAG AAG ACT GCT TTT CCA CCA A |
| NaP-3R2 | CAG GGG TGC ATG ATT CAT ATT |
| NaP-3R3 | GCC CAT CCT TGA GAA CTT GTT A |
| *Primers for GFP constructs* |  |
| n-NaAgfp-1p | AGC TCT AGA ATG TGG AGG ATT GCC AAA AAA T |
| n-NaAgfp-2m | AGC GTC GAC GCG AAG ATG AAG CTA ACG ACG A |
| n-NaAgfp-3p | AGC TCT AGA CAT GGC TTC TAT CTC CTC TGG C |
| NaA_mut_p | ACG ATC TGG AGG ATT GCC |
| NaA_mut_m | CAA TCC TCC AGA TCG TTT T |
| n-NaCgfp-1p | AGC TCT AGA ATG TGG CGG TTT GCC AAA AAA |
| n-NaCgfp-2m | AGC GTC GAC GCA ACG ACG AAG CTA GCG A |
| n-NaCgfp-3p | AGC ACT AGT GGC CAT CTC CCT CAT CTC TCA |
| NaC_mut_p1 | ATC CTG AAA ATT ATC TGG CGG TT |
| NaC_mut_m1 | AAC CGC CAG ATA ATT TTC AGG AT |
| NaPgfp-1p | AGC TCT AGA CTG GCT TCA ACG GCC GCA GC |
| NaPgfp-2m | AGC GTC GAC GGT TGG TCT GAA TGC ATC TG |
| NaPgfp-3p | AGC TCT AGA ACC ATC TCC ATC TCT CCA CTT C |
| NaPgfp-mut-p | CCT TCC ACC ACG GCT TCA ACG |
| NaPgfp-mut-m | CGT TGA AGC CGT GGT GGA AGG |
| NaAI-9m | TCT CTT CCC AGG GCT GCA TAG T |
| NaAI-10p | GGC TCT TGT AAT GGC TTG CAA C |
| NaAI-10m | TCA GCA ATT CCG GAG TAA ACA T |
| NaAI-11p | GAA CCT GGT TGC TGG GGA GAA G |
| NaAI-11m | GTG CAG CAC TTG GGT TAT TTG T |
| NaAI-12p | ATC CTA CAA ATA ACC CAA GTG C |
| NaAI-12m | GAA TGG CAT CCC GCT CCT TTA G |
| NaAI-13p | CTA AAG GAG CGG GAT GCC ATT C |
| NaAI-13m | ATC CCC CAG CCA GCT CAT AAT G |
| NaAI-14p | TGA CTG CCT TGG AGG AGA TGT T |
| NaAI-14m | AAG GGG AGT AGT CCA CCT CAC A |
| NaAI-15p | GTG AGG TGG ACT ACT CCC CTT G |
| NaAI-15m | TTT TAA CGC CAA AAC CTG AAG A |
| NaAI-16p | CTC TTC AGG TTT TGG CGT TAA A |
| NaAI-16m | ATC ATG TGA GAT CCA TCG AGG G |
| NaAI-17p | CCC AAA TTT TGT ACA TTC CCT C |
| NaAI-17m | TCG GCT GCT CAT AGA GTT CCA C |
| NaAI-18p | ACT GAG GGA GAA ATT TGT GGA A |
| NaAI-18m | GAT CCG GAA GAG GAG GAA AAC T |
| NaAI-10p5 | CCA GCC GAG TCT GAC ACT |
| NaAI-10p6 | GAC CTA GTC CAA CGG CTA CAA G |
| NaAI-11pm4 | TTT AAT TTG GCT TTG GTT TAG G |
| NaAI-11pm5 | GAG CTT TTT CTC CAA TCA TCC A |
| NaAI-5p3 | TCA TAT GCG GAA AGG GGT TGT A |
| NaAI-5m2n | GCA GCG AGA GGT TTG GTA AAG T |
| NaAI-6p3 | TCC AGT CCA CGG CAC CAC GAC |
| NaAI-10p2 | TGC TTC CTA AAT ACC AAA ACC T |
| NaAI-11p2n | TGC TTA TAA TAT GCC AGA TTT G |
| NaAI-11m3 | CAA AAT GGA CTC GGC TCA ACT T |
| NaAI-15m3 | ATT TCT TAT CTA GCC CTC ACC A |
| NaAI-5p2 | CAT TAT AAA TAT GGG GCA AAG C |
| NaAI-5m2 | TCT GGG CTT GGC TCA GTC TCA T |
| NaAI-6p2 | CCG ATA TGA TAA ACT CAG AAA A |
| NaAI-11p2 | ATA TGC CAG ATT TGT CTC TAC A |
| NaAI-11m2 | ATT TAC ACC GTT ACT AAA GCA G |
| NaAI-15m2 | TGC AAT AGA AAT CCC GAA TGA T |
| NaAI-4m-p | GGC AAC TTG ACC CAG CAG AGT A |
| NaAI-5p3 | TCA TAT GCG GAA AGG GGT TGT A |
| NaAI-6p3 | ATC CAG TCC ACG GCA CCA CGA C |
| NaAI-seq7 | CAC AAA CTC GGC TAT AAG GTC G |
| NaAI-10p2 | CAT GGA CCA CCT GCT TCC TAA A |
| NaAI-11p-m | GCC CAT TTT AGT TAC ACA CTG A |
| NaAI-11p2 | TCA GAT ATT GAC CTC AAA TCA T |
| NaAI-15m3 | AAT TTC TTA TCT AGC CCT CAC C |
| NaAI-18m-p | GTG GGT GAG TTA GCT TCC ATC A |
| NaAI-5n1p1 | TCA GAT GTA TGA GGG TCC GCA C |
| NaAI-10p6 | GAC CTA GTC CAA CGG CTA CAA G |
| NaAI-11pm5 | GAG CTT TTT CTC CAA TCA TCC A |
| NaAI-5n3p | CAG ATG TAT GAG GGT CCG CAC |
| NaAI-5n3m | GAG TCT GGG CTT GGC TCA GGT C |
| NaAI-5p5 | GAA TGT ATT TAC CGC TGA TGG A |
| NaAI-10p4 | TTA ACA CTT CGA TTA AAC ATG A |
| NaAI-11p-m3 | ATC TGA ACT TTA TCC AAG ACC C |
| NaAI-1m3 | AAG TAG ATT GGG CGA GAG GTT C |
| NaAI-5p4 | CCA TAG AAG AGA GCA TAG AAA A |
| NaAI-6p4 | TTT CCG TTT ACT GTT CGT GTT C |
| NaAI-10p3 | GCT AAA GGC TCG CAC AAC CTA C |
| NaAI-11p-m2 | AGG ACA AAA GGC AAA ACT AGA A |
| NaAI-11p3 | GTG CAC CCT TGT ACA TAG ATA A |
| NaAI-15m4 | CGC ATC TAT CTA CTG AAG TTT T |
| Na_seqA_seqII | CTT TAA TGC TTC GTG CTA ATG C |
| Na-seqA-seq3 | GGT ATT AGA GCG GGA CAC AAG |
| Na-seqA-rev2 | TTG GGT CAT TCT ATC CTT AAC |
| Na-seqA-seq4 | ATT TGT GCC AAA AAC TCA AGG A |
| Na-seqA-rev3 | CAT GCT GTT AGT TCT TCG TTG G |
| Na_seqA_seq5 | GGC AAT TAT CAA AAT GTG ACC T |
| Na_A_seq5 | CTT CTA ACA ATG GCA ATT ATC A |
| Na_A_revD | AAC TTG CCT TGA GAG AGT GGT C |
| Na_seqA_seq6 | TGG CCA AAA TCA AGA CAT GAA T |
| Na_seqA_revE | GAT GCC ATG AAG GTA AAG AGG A |
| NaCI1p | TCA TGG TTG CGC TAG GGT TGT C |
| NaCI1m | GGC GCT GAA TCA TCC TTT CCT T |
| NaCI2p | CGG CAG GTG ACT AAG TTA GTG A |
| NaCI2m | ATA AGC CGT TTC AAT CAA CAA C |
| NaCI3p | GGA TGG TCC ACC TGA TAT TCG T |
| NaCI3m | TTT ATC AAG GCC CTT ACG AAC T |
| NaCI4p | ATG GTG TTA TTG AAT GTG ATC C |
| NaCI4m | GTG GCA CCA ACA TTG GCA TGT A |
| NaCI5p | GAT TCC TTA CAT GCC AAT GTT G |
| NaCI5m | GCT GTT GCC TTG ATC CAT GAG T |
| NaCI6p | GCA CTC ATG GAT CAA GGC AAC A |
| NaCI9m | TCT TCC CAG GGC TGC ATA ATG T |
| NaCI10p | GTT TGG GAA TTT GGG TTA TGC C |
| NaCI10m | AAC ATC TGC GGG TTT CTC ACC A |
| NaCI11m | ATC AAA AGC CTT GCA CGC ACA G |
| NaCI12p | ATC ATG CGA AGG GAT TCA CAA A |
| NaCI12m | GAA TGA CAT CCC GCT CCT TTA G |
| NaCI13p | CTA AAG GAG CGG GAT GTC ATT C |
| NaCI13m | ACA ATC ACC CAG CCA GCT CAT A |
| NaCI14p | CTG CCC TGG AGG AGA TGT TTC A |
| NaCI14m | CAA GGG GAG TGG TCC ACC TCA C |
| NaCI15p | GTG AGG TGG ACC ACT CCC CTT G |
| NaCI16m | TTT GCA GGC AAT AGC TGT CAT C |
| NaCI17p | ACT GCT TTT CCC CCT AAT TTT G |
| NaCI17m | AAT CGG CTG TTC ATA GAG TTC C |
| NaCI18p | ATG CGA TGT GGA TGA AAT GAA C |
| NaCI18m | ACG GTC TGG AAG AGG AGG AAA A |
| Na_seqC_seqI | CCA CTG GCC CAT ATT CTC TCT A |
| Na_BAC2_seq2m | ATA TCG GCT GAT CTT AAA TC |
| Na_BAC2_seq3m | GAA GGT TCA AGG TGG TGG TTA G |
| Na_BAC2_seq7 | CCG CAC CCG AGT CAT GTC AC |
| Na_BAC2_rev2 | GGC CGC ATC GAA TAT AAC TT |
| Na-BAC2-seq8 | TCC TTA AGC TGG TAC AAG AAT A |
| Na-BAC2-seq9 | GAG GGT GTT ATG AGC AAG ATG G |
| Na-BAC2-rev4 | CAA TTA TGC TTT CTG GCC TGT A |
| Na_seqC_rev5 | AGC AGC TTT ACG ACG GCG ACT C |
| Na_seqC_rev7 | ATC CCG AAT TTG CTC CTC CAT C |
| Na_BAC2_seq10 | GGT CTA GAT CCA TGT ATG GTA A |
| NaCI-14p2 | GAA GGT TTA CAA TCA CGC AGA G |
| NaCI-11p2 | ATA GAA ACC CAA TGT TAT CGT |
| NaCI-11m2 | TAT GTT ATG TTG CCT CCT AAG |
| seqC-rev6m | AAG TTT GGG AAT TTG GGT TAT G |
| NaCI-1pm | GCA AAA GAT GGC AGA CCT CCT A |
| NaCI-11pm | CTA GTG ATT CAG ATG GCA AGT |
| NaCI-4p2 | ATG TTT CGA CTC CAG CTT GAT G |
| NaCI-rev6 | TGC AGA CAG GTT CCC AAA TAG A |
| NaCI-1pm | AGA GAA CTT GCC CAC CTC GTT T |
| NaCI-5mp | CAG CTA TGA CAA GGG TGC ACA T |
| NaCI-rev6 | TCC GGA TCT GAC CTT TAC CAA C |
| NaCI-seq9 | CAT TTG GAT CCT GGA CAT TGG |
| NaCI-11pm | GGA TGC AGT AGT GGC GTA GCT C |
| NaCI_11pm2 | CAT TAA ACA AAC CAA ATC AGG T |
| NaCI_11p3 | CAA TCC CTC ATT ATC ATT TCT T |
| NaCI_11m3 | CCT TCA AAA GTT GTA CTA AAG A |
| NaCI_14p3 | TGC ATC TTC CTT AGA TTT GGT T |
| NaCI-15pm | CCT CTG CCT CTT AAT CAT GAC C |
| NaCI_15pm2 | GAT TTA TTC TTA AAC AGG ACC A |
| NaCI_18p2 | CAA TAA CCA GAT TTT TCC CAA T |
| NaPI-1p | TGA ATG GGC AAG ACG ATG |
| NaPI-1m | AAT TTG GTT TTC CCT TGT GGA T |
| NaPI-2p | CAA AAA TCA GGA GGC TCT CA |
| NaPI-2m | ATT AAG TGG AGG TTG CAC ATA A |
| NaPI-3p | AAA CAG CTT ATG TGC AAC CTC C |
| NaPI-3m | CAA GCC CTT TAC GTA CAA GTG |
| NaPI-4p | TGA TCC ACT TGT ACG TAA AGG G |
| NaPI-4m | TTT GGT GGT ATC AAC ATC GG |
| NaPI-5p | CAT GCC GAT GTT GAT ACC AC |
| NaPI-5m | CGT TGT TGC TTT GCT CCG TG |
| NaPI-5m2 | GTT GTT GCT TTG CTC CGT GA |
| NaPI-6p | TAT GTT ATG CGC ACT CAC GGA G |
| NaPI-6p2 | ACA CCC GCC GCG CTT AAT GC |
| NaPI_6p3 | TCA TGT AAC TCG CCT TGA TCG T |
| NaPI_6p4 | CTC TAG GCC GCG ATT AAA TTC C |
| NaPI_6p5 | ACC GGA TTC AGT CGT CAC TCA T |
| NaPI_6p6 | CGG GTT GGA CTC AAG ACG ATA G |
| NaPI-6m | GCC CGC TTG CCC ATA AG |
| NaPI-7p | GGC AAG CGG GCG ACC TAT T |
| NaPI-7m | TGA CAC TCC ACC GCC ACC GT |
| NaPI-8p | GAC GGT GGC GGT GGA GTG TC |
| NaPI-8m | ATA GGC ACG CCC ACG AAA |
| NaPI-9p | TTG ATT CGG CAG AAA GAC CTA T |
| NaPI-9m | GCC CCA AAG CTG CAT AAT G |
| NaPI-10p | TCG TGC AAT GGT TTA CAG CAT |
| NaPI-10m | TCT CTT CTT GCA GCT ATA CCC |
| NaPI-11p | CGG CTT CAG TGA ACC TAG TT |
| NaPI-11m | AAG TTG GAT GCA CAG CAG GAT |
| NaPI-12p | CCA ACT TCT CTA CTT GCT CGT A |
| NaPI-12m | CAA GCT GCA CTA AAT AGG ATC T |
| NaPI-13p | AAA GAA GAT TGA AAG AGC GTG A |
| NaPI-13m | GCC AAG ACA TAA TAC TAC GTG C |
| NaPI-14p | GAG ATG TTC CAA GCT GCA CGT A |
| NaPI-14m | ACT GTT TCC AGT ATG GCT GCA C |
| NaPI-15p | TGG GCT TCC AGT TGT GCA G |
| NaPI-15m | TGC TCT CCC TCT GTA ATG CCA G |
| NaPI-16p | CCC TTC AGG TTC TGG CAT TAC |
| NaPI-16m | TAT GTG AAC CAT CAA GGG AGT G |
| NaPI-17p | CCA CCA AAC TTT GTT CAC TCC C |
| NaPI-17m | GGC ATG CTA TAA AGC TCA ACG A |
| NaPI-18p | TGA TGA TAT GAG CCG CAT TC |
| NaPI-18m | CGC CAC CTT GAA AGT AAT CCC |
| NaPI-18m2 | TCA GAT CAA AAT CAC CAC GG |
