# Supplementary material for: Evolution of plant phage-type RNA polymerases: the genome of the basal angiosperm Nuphar advena encodes two mitochondrial and one plastid phage-type RNA polymerases
Source: BMC Evol Biol. 2010 Dec 6;10:379. doi: 10.1186/1471-2148-10-379 (PMC3022604; doi:10.1186/1471-2148-10-379)
Supplement: Additional file 2 — Merged conserved blocks of 41 RpoT sequences used for reconstruction of phylogeny. [file 1471-2148-10-379-S2.DOC]

**Additional file 2 – Merged conserved blocks of 41 RpoT sequences used for reconstruction of phylogeny**

1 10 20 30 40 50 60

| | | | | | |

PotRpoTp1 YNLLRRRQIREETEAWERMADEYRGLVREMCERKLAPNLPYVKGLLLGWFEPLKEAILPA

PotRpoTp2 YNLLRRRQIREETEAWERMAEEYTGLVKEMCERKLAPNLPYVKSLFLGWFEPLKEAILPA

VvRpoTp YGLLRRRQVKAETEAWERMVEEYREIEREMCEKKLAPNLPYVKALFLGWFEPLREAILPA

NsRpoTp YAMLRRRQIKAETEAWEQMVEEYRELEREMCEKKLAPNLPYVKKLLLGWFEPLRQAILPA

AtRpoTp FDSLRRRQVKEETEAWERMVDEYRDLEKEMCEKNLAPNLPYVKHMFLGWFQPLKDVILPA

SoRpoTp YNLLRRRQIKAETEAWESMVEEYREFVREMRDKKLAPNLPQVKALFLGWFEPLKKAILPA

ZmRpoTp LRRLRQRQVKAETEAWARAAEEYREIEREMLDRRLAPALPYVKALFVGWFEPLRDAILPA

SbRpoTp HRRLRQRQVKAETEAWARAAEEYREIEREMLDRRLAPALPYVKSLFVGWFEPLRDAILPA

OsRpoTp HRRLRQRQVKAETEAWARAADEYRELEREMLDRRLAPALPYVKSLFLGWFEPLRDAILPA

HvRpoTp HRRLRQRQVKAETEAWARAAEEYRQLEREMLDRNLAPQLPYVKSLFLGWFEPLRDAVLPP

TaRpoTp HRRLRQRQVKAETEAWARAAEEYRQLEGEMLDRHLAPQLPYVKSLFLGWFEPLRDAVLPA

SbRpoT1 YTSLRRRQIRVETEAWEQAAKEYRELLADMCEQKLAPNLPYIKSLFLGWFEPLRDQILPA

ZmRpoTm YTSLRRRQIRIETEAWEQAANEYRELLADMCEQKLAPNLPYVKSLFLGWFEPLRDQILPA

HvRpoTm YVSLRQRQIRIETEAWEQAAKEYRELLADMCEHKLAPNLPYVKSLFLGWFEPLRDQILPA

TaRpoTm YVSLRQRQIRIETEAWEQAAKEYRELLADMCEHKLAPNLPYVKSLFLSWFEPLRDQILPA

OsRpoTm YAMLRRRQIQIETEAWEQAAEEYRELLADMCQQKLAPNLPYVKSLFLGWFEPLRDQILPA

NaRpoTm2 YNALKRRQIKIETEAWEQAAKEYKELMMDMCKNKLAPNLPYIKSLFLGWFEPLRDRILPA

NaRpoTm1 YKAQKRRQIKIETEAWEQAAREYRELFVDMCKQKLAPNLPYMKSLFLGWFEPLRDRILPA

VvRpoTm HNVLRRRQIKMETEAWEEAAKEYRELLSDMCEQKLAPNLPYIKSLFLGWFEPLRDVILPA

NsRpoTm YNLLRRRQIKVETEAWEEAAKEYQELLMDMCEQKLAPNLPYMKSLFLGWFEPLRDAILPA

PotRpoTm1 YAILKRRQIKMETEAWEQAAQEYQEMLEDMCEQKLAPNLPYVKSLFLGWFEPLRDAILPA

PotRpoTm2 YTILKRRQVKMETEAWEQTAREYQEMLEDMCEQKLAPNLPYVKSLFLGWFEPLRDAILPA

RcRpoTm YTILKKRQIKMETEAWEEAAREYQELLTDMCEQKLAPNLPYIKSLFLGWFEPLRDAILPA

CaRpoTm YNVLRKRQIKVETEAWNEAAREYQELLADMCEQKLAPNLPYMKSLFLGWFEPLKDAILPA

NsRpoTmp YQTLKRRQVKVETEAWEQAAKEYKELLFDMCEQKLAPNLPYVKSLFLGWFEPLRDKILPA

VvRpoTmp YLRLRRRQVKIETEAWELAAKEYKELLMDMCEQKLAPNLPYMKSLFLGWFEPLRDAILPA

PotRpoTmp YKALRRRQVKIEAEAWEQAAKEYKELLKDMCEHKLAPNLPYMKGLFLGWFEPLRDAILPA

AtRpoTmp FQNLWRRQVKIETEEWERAAAEYMELLTDMCEQKLAPNLPYVKSLFLGWFEPLRDAILPA

CsRpoTmp YQSLKRRQVKIETEAWEKAANEYKELLTDMCEQKLAPNLPYVKSLFLGWFEPLREAILPA

SoRpoTmp YQKLRNRQIKLETESWQQAANEYKELLADMCKLKLAPNLPYVKSLFLGWFEPFRDSILPA

AtRpoTm FYMLKQRQVKMETEEWERAARECREILADMCEQKLAPNLPYMKSLFLGWFEPVRNAILPA

BoRpoTm YYSLKQRQVKLETEEWEKAAKECQEIIEDMCEQKLAPNLPYVKSLFLGWFEPLRDAILPA

NaRpoTp YNLLRRRQVKMETEAWEDAAREYRELIKEMCEKKLAPHLPYVKSLFLGWFEPFRDAILPA

PpRpoTmp1 EKALRMRQFKIETEAWHQAAAEYKELVAEMCKKNLAPNLPATRSLLLGWFEPLRDAILPA

PpRpoTmp2 ARELHKRQVKIETEAWQQAATEYRELMTEMCRKSLAPNLPFAQSLLLSWFEPLRDGILPA

PpRpoTm LQELRQRQIINETEAWTNAEAQYEEFIAEMCRKKLAPNLPASQLLLLGWYEPLRDAILPP

SmRpoTm ARRLFNRQQKLELDAWDAAVREYRKILVEMCRKKLAPNLPFAKSLMVSWFEPVRDEILTA

MspecRpoT MRELEERQRRNEILAVDREVERYKEEARITRRRGGGAELPLGRRLLQGFFAPLTSAILNP

MipuRpoT ------------------MVEHAREAA---SRGDGLGATAAGRNFLHNFLEPLSRAILNP

OlRpoT RRAQTRKQREAENLAVEREVMRYKELARKTFAAGIGAQLPVVQKLLASFYVPLVEALLEP

OtRpoT EQLQLRKQREAENLACEREVARYKEFARKTFAAGIGAQLPVVQKLLASFYVPLVDAILEP

PotRpoTp1 DKMAVIVMHKMMGLLMVGGCVRVVQAAVQIGMAIEQEVRIHNFLEKTKNYEKEVLRKRVN

PotRpoTp2 DKMAVIVMHKMMGLVMVGGCVRVVQAAVQIGIAIEQEVRIHNFLEKTKNHEKEMLRKRVN

VvRpoTp DKMAVIVMHKMMGLVMMGGCVRVVQAAVQIGMAIEQEVRIHSFLEKTKNFEKEILRKRVK

NsRpoTp DKMAVIVMHKLMGLLMMGRCVQVVQAAVQIGMAVENEVRIHNFLEKTKKLETMILRKRVK

AtRpoTp DKMAVIVMHKMMGLVMSGGCIQVVQAAVSIGIAIEQEVRIHNFLKRTRKNEKQLLRKRVN

SoRpoTp DKMAVIVMHKMMSLLMVGGCVRLVQAALQIGTAIEQEIKIHSFLEKTKNIEKEIQRNRVK

ZmRpoTp DKVAVIVMHKMMGLLMSSGSVRVVQAAHCIGEAVEREFKVQSFFQKSRKKEQAKCRKRVK

SbRpoTp DKVAVIVMHKMMGLLMSSGSVRVVQAAHCIGEAVEREFKVQSFFQKSRKKEQAKCRKRVK

OsRpoTp DKVAVIVMHKMMGLLMSSASVRVVQAAHCIGEAVEREFKVQTFFQKTRKKEQAKCRKRVK

HvRpoTp EKVAVIVMHKMMGLLMSSGSVRVVQAAHSIGEAVEREYKVQAFFQKTRRKEQAKCRKLVK

TaRpoTp EKVAVIVMHKMMGLLMSSGSVRVVQAAHSIGEAVEREYKVQAFFQKTRRKEQAKCRKLVK

SbRpoT1 DMMAVITMHKLMGLLMTGGSVRVIQAACQIGEAIEHEVRINRFLEKTRKKEQQRLRKKVT

ZmRpoTm DMMAVITMHKLMGLLMTGGSVRVIQAACQIGEAIEHEVRINRFLEKTRKKEQQRLRKKVT

HvRpoTm DMMAVITMHKLMGLLMTGGSVRVIQAACQIGEAVEHEVRIHKFLEKTKKKEQQLLRKKVT

TaRpoTm DMMAVITMHKLMGLLMTGGSVRVIQAACQIGEAVEHEVRIHKFLEKTKKKEQERLRKKVT

OsRpoTm DMMAVITMHKLMGLLMTGGSVRVIQAACQIGEAIEHEVRIHKFLEKTKKKEQERLRKKVT

NaRpoTm2 EMMAVITMHKLVGLMMTSGCARVVQAACRIGDAIENESRIHSFLEKTKKKEKEFLRKKVT

NaRpoTm1 DMVAVITMHKLMGLMMTSGCARVVQAACQIGEAIENEARIHRFLEKTKKKEKEFLRKKVT

VvRpoTm DMMAVITMHKLMGLLMTGGSARVVQAACHIGEAIEHEVRIQRFMEKTKKKEQEKLRKKVS

NsRpoTm EMMAVITMHKLMGLLMTGGSARVVQAASHIGEAIEHEARIHRFLEKTKKSERERLRKKVK

PotRpoTm1 DMMAVITMHKLMGLLMTGASIRVVQAASVVGEAIEHEGRIHKFLEKTKKREQEKLRKKVT

PotRpoTm2 DMMAVITMHKLMGLLMTGASIRVVQAASVVGEAIENEAKIHKFLEKTKKREQEKLRKKVT

RcRpoTm DMMAVITMHKLMGLLMTTGSVRVVQAACAVGEAIEHEARIHRFLEKTKKKEQEKLRKKVT

CaRpoTm DMMAVITMHKLMGLLMTGGGTTVIQAATQIGEAVEQEAKIRKFLEKSKKKAQQRLAKKVT

NsRpoTmp DMMAVITMHKLMGLLMTGGTARVVQAALVIGDAIEQEVRIHNFLEKTKKQEQEKLRKKVT

VvRpoTmp DMMSVITMHKLVGLLMTGGSTRVVQAACVIGDAIEQEVRIHNFLEKTKRKEQEKLRKKVT

PotRpoTmp DMMSVITMHKLTAMVMIGGCARVVAAACMIGDAIEQEIRIHNFLEKTRKKEEEKLRKKVT

AtRpoTmp DKISVITMHKLMGHLMTGGCVKVVHAACTVGDAIEQEIRICTFLDKKKKGEQDKLRKKVN

CsRpoTmp DMIAVITMHKLMGLLMTGGCVKVIMAACTVGDAIEQEIRICRFLEKTKKKEEDWLKRKVS

SoRpoTmp DMMAIITMHKLMGMLMTGGSVKVVQAACALGEAIEQEVRIHTFLEKAKKKEQQHLRKNVN

AtRpoTm DKMAVITMHKMMGLLMTNGIVKLVNAATQIGEAVEQEVRINSFLQK-KNKETEKARKQVT

BoRpoTm DMMAVITMHKMMGLLMTNGVVKVVNAATQIGEAIEQEARITSFMKKAKKKETLKLKKQIN

NaRpoTp DMMAVIVMHKMMVLLMNGGCVRLVHAACHIGEAIEQEVRIYNFFQKIKKSNQEALRKRVT

PpRpoTmp1 DMLAVITMHRLMSLLMSDGCVKVVHAALQIGEAVEQEVGIYKLLRSKRKV-LKLAKEKVK

PpRpoTmp2 DMLAVITMHRLMGLLMCDGCVKVIHAAVVIGEAVEQEVRIFQLMNSQKKSSKKVVRDKVK

PpRpoTm SQLAVITMHCLLALVMSNGYVKVIQAALHIGEAVEQEVLIRKLRIGKAKKTSPELPTQAT

SmRpoTm DVLAVITMHRLVALMMQDGCIRLANTAVLIGDAVEQEIKIRRALKKRKPKKKEYLKDKLR

MspecRpoT DKLAVITLHTVISQLMKGGMCKFVRAVEMLGQSVQAEVNLARLRQRARLTRFMKVN--ME

MipuRpoT DKLAVITLHAVISQLMMGGKSKFVRVVERLGEAVQAEVNLSRLKQRGKVRETAGVS--AK

OlRpoT DKLAVLTLHATLSTLMKGGAAKFISVADQVGSAVQAEVNLERMRSAEKAAAAEHRINLAL

OtRpoT EKLAVLTLHTTLSTLMKGGSAKFITVAEQVGIVVQAEVNLGRMRAAEKIALVEQRINLTL

PotRpoTp1 SLIRRKRLMEVQNLVKWSRGTQAKLGSRLIELLTETAYVQPPPDVRPAFRHIFKTLTKNP

PotRpoTp2 SLIRRKRLMEVQNLVIWNRDKQAKLGSRLIELLTETAYVQPPPDVRPAFRHIFKSVTKNP

VvRpoTp SFIRKKKIIEVQKLVKWGRDTQAKLGSRLIELLTETAYVQTPPDVRPAFRHTFKTVTKES

NsRpoTp SLIKRNRVVEVRKLMKWGRDTQAKLGCRLLELLTETAYVQPPPDIRPAFRHVFRIATRDP

AtRpoTp SLIRRKRIIDALKVVKWGRATQAKLGSRLLELLIEAAYVQPPPEFRPAFRHRFKTVTKYP

SoRpoTp SLIKRKRLFEAAKLVTWSQDTQAKLGSRLIELLIDTAYIQHPPDVRPAFRHKFKILNNEL

ZmRpoTp SLVKRRKMSEAQRLVQWGTEAQVKLGSRLIELLLDSAFVQPPPDIRPAFKHVLRQPIIEN

SbRpoTp TLVKRRKMSEAQKLVQWGTEAQVKLGSRLIELLLDSAFVQPPPDIRPAFKHVLRQPIIEN

OsRpoTp SLVRRRKLTEAQKIVQWGTESQVKLGTRLIELLLDSAFVQSPPDIRPAFKHVLRQPIVEN

HvRpoTp SLVRRRKLTEAQKLVQWGAEAQVKLGTRLIELLLDSAFVQSPPDFRPAFKHVLRKPIVEN

TaRpoTp SLVRRRKSTEAQKLVQWGTEAQVKLGTRLIELLLDSAFVQSPPDFRPAFKHVLRKPIVEN

SbRpoT1 DLMKKQKLRQVRNIVKWGQDVQAKVGSRLIELFMDTAHIQPPPDIRPAFRHEMRTVAKEQ

ZmRpoTm DLMKKQKLRQVRNIVKWGQDAHAKVGSRLIDLFIGTAHIQPPPDIRPAFRHEMRTMVKEQ

HvRpoTm DLMKKQKIRQVRHLVKWGQDAHAKVGSRLIELFIETAHIQPPPEIRPAFTHEMRTVAREQ

TaRpoTm DLMKKQKIRQVRHLVKWGQDAHAKVGSRLIELFIETAHIQPPPEIRPAFTHEMRTVAREQ

OsRpoTm DLMKKQKIRQVRNIVKWGQDAHAKVGSRLIELMIETAYIQPPPDIRPAFTHEMRTVAREQ

NaRpoTm2 NLIKKQKLRQVTKLVKWGVEAHAKVGCHLIELLIETAYIQPPPDIRPAFRHSLRNVSKEQ

NaRpoTm1 SLIKKQKLRQVTKIVKWGTEGHAKVGCRLIELLMK-PLISAPHLYSSAFRHSLRSPSNHQ

VvRpoTm MLMKKQKLQQVRQIVKWGQDANVKVGSRLIELLIETAYIQPPPDIRPAFVHTLKTVIKET

NsRpoTm ILMKKQKLQQVRKIVKWGQDNLVKVGCRLIQILMETAYIQPPPDIRPAFVHTLKTV--ET

PotRpoTm1 TLIKKQKVQQVRRIVKWGQEEHVKVGSRLIQLMIETAYIQPPPDIRPAFVHTLKTITKDT

PotRpoTm2 TLMKKQKVHQVRRIVKWGQEAHLKVGSRLIQLMIETAYIQPPPDIRPAFVHTLKTITKDT

RcRpoTm ELMKKQKVQQVRGLTKWGQEAQVKVGCRLIQLLIETAYIQPPPDIRPAFMHTLKNVMKDT

CaRpoTm TLMKKQKISQVREIVRWSQDNQVKVGCKLIQLLAETAYIQPPPDIRPAFIHTQKTVCKES

NsRpoTmp NLMKKQKLRAVGQIVRWGQDARAKVGSRLIDLLLQTAYIQPPPDIRPAFVHSVRTVAKET

VvRpoTmp NLMKKQKLHAARQIVKWGPEAKAKVGSRLIELLMQTAYIQPPPDIRPAFVHTFKTVMREA

PotRpoTmp DLIKKQKLPAVRKIVKWSTDAKAKVGSRLIELLLQTAYIQPPPDLRPAFVHTFRTVSYEN

AtRpoTmp ELIKKQKLSAVRKILQWIADVRAKVGSRLIELLVRTAYIQSPPDVRPAFVHTFKVAKG-S

CsRpoTmp ELIKKQKLPAVRQILNWSTDVRAKVGSRLIELFMKTAYIQPPPDIRPAFVHTFKTVKN-S

SoRpoTmp NLIKKQKRRQALKIVDWGSETRAKVGSRLIELLIQTAYIQPPPDLRPAFVHSLKSVGPEN

AtRpoTm VLMEKNKLRQVKALVRWGQEAQVKVGARLIQLLMENAYIQPPPDIRPAFKQNFRTVTLEN

BoRpoTm TLLKKHKVRQVREIVKWGQEAQVKVGARLIQLLMETAYIQPPPEIRPAFKQSSRIVTIEN

NaRpoTp NLLKKKKIRDVQLLVEWGRDSHAKLGSRLIDLLIQTAYVQPPPEIRPAFRHTFKTIKKEP

PpRpoTmp1 KLVKQQKLRLVGKVVQWGPAIQVKVGSRLLELMLETSVMRSPTELRPAFKHTLRNYPIKN

PpRpoTmp2 KLVKQNKLRRVDSILKWSTVIHVKLGSRLLELMLETSFVRAPGELRPAFQHKFKNHVLR-

PpRpoTm GMQKLRKLRLWRSMLQWGPTIYAKVGSRLLELFMETAVIRVPASFEPVFQHTNKKFVCTS

SmRpoTm RAIIE-RPRNVKEILKWPSFIQAKLGCRLIDIMMSNSHINVPTEIRPAFRHVLKVP----

MspecRpoT SLKTLTTVRGVARLARWGGVTRSKVGAALVKLLLTTARIRVPLIEVPAFYHDYVKH-KAH

MipuRpoT SLSKLTTVRSVAKFARWGMTARVKLGSVLAKLLVETAKIEVPLEIKPAFEHHYEYVNRQQ

OlRpoT DTSKMNTIKSVSKHARWGREIRLKIGTVLLTALMNTAKIGVPLLTLPAFYHDYKEA----

OtRpoT DTSKMNTIKSVSKHARWGREIRVKVGTVLLNALMNTAKIGVPVLTLPAFYHDYREG----

PotRpoTp1 GQKIVKKYGVIECDPLILTGLDGTAKHMLIPYFPMLVPPKKWKGYDKGGHLFLPSYVMR-

PotRpoTp2 GQKTVKKYGVIECDPLILTGLDGTAKHMLIPYFPMLVPPKKWRGYDKGGHLFLPSYIMR-

VvRpoTp GQKDWRKYGVIECDPLVLIGLDRTAKHMVIPYVPMLIPPKKWKGYDKGGYLFLPSYVMR-

NsRpoTp GKSIVKKYGVIECDPLVVAGVDRTVKQMMIPYVPMLVPPKKWRGYDKGGYLFLPSYLMR-

AtRpoTp GSKLVRRYGVIECDSLLLAGLDKSAKHMLIPYVPMLVPPKRWKGYDKGGYLFLPSYIMR-

SoRpoTp GQQVVKRYGVIECDPLVLKGIDRSARYMIIPYVPMLIPPKNWKGYDKGGYLFLPSYVMR-

ZmRpoTp G-RLKKKHWVIECDHLVHEGFESTARHVDIPYLPMLVPPKKWKGYDKGGHLFLPSYIMR-

SbRpoTp G-RLKKKHWVIECDHLVHDGFESTARHVDIPYLPMLVPPKKWKGYNKGGHLFLPSYIMR-

OsRpoTp G-RLKKKHWVIECDPLVHEGFESTARHVEIPYLPMLVTPKKWKGYDTGGYLFLPSYIMR-

HvRpoTp G-RLKKKHFVIECDPLVHEGFESTARHVEIPYLPMLVPPTKWKGYDKGGHLFLPSYVMR-

TaRpoTp G-RLKKKHFVIECDPLVHEGFESTARHVEIPYLPMLVPPTKWKGYDKGGHLFLPSYVMR-

SbRpoT1 QKSSRR-YGVIKCDPLVRQGLDRTAKHMVIPYMPMLIPPICWTGYDKGAHLFLPSYVMR-

ZmRpoTm QKSSRR-YGVIKCDPLVRQGLDRTAKHMVIPYMPMLIPPICWTGYDKGAHLFLPSYVMR-

HvRpoTm --KSRR-YGVIKCDPLVRQGLDRTAKHMVIPYMPMLIPPINWTGYDKGAHLFLPSYVMR-

TaRpoTm R-KSRR-YGVIKCDPLVRQGLDRTAKHMVIPYMPMLIPPINWTGYDKGAHLFLPSYVMR-

OsRpoTm QKSSRR-YGVIKCDPLVRQGLDRTAKHMVIPYMPMLIPPISWTGYDKGAHLFLPSYVMR-

NaRpoTm2 SSNSRR-YGVIECDPLVRKGLDKTARHTVIPYMPMLVPPLCWTGYDKGAHLFLPSYIMR-

NaRpoTm1 QNNSRR-YGVIECDPLVRKGLDRTARHMVIPYMPMLVPPLGWTGYDKGAHLFLPSYVMR-

VvRpoTm QKGTRR-YGVIECDPLVRKGLEKTARHMVIPYMPMLVPPLNWTGYDKGAYLFLPSYVMR-

NsRpoTm MKGSRR-YGVIQCDPLVRKGLDKTARHMVIPYMPMLVPPQSWLGYDKGAYLFLPSYIMR-

PotRpoTm1 QKSSRR-YGVIECDPLVRKGLEKSARHMVIPYMPMLVPPLNWTGYDQGAHLFLPSYVMR-

PotRpoTm2 QKSSRR-YGVIECDPLVRKGLEKSARHMVIPYMPMLVPPLNWTGYDQGAYFFLPSYVMR-

RcRpoTm QKTSRR-YGVIECDPIVRNGLEKSARHMVIPYMPMLVPPLNWEGYDQGAYLFLPSYVMR-

CaRpoTm QKGLRRRYGVIECDPLVRRGLEKTARHMVIPYMPMLIPPLNWTGYDKGAYLYLPSYIMR-

NsRpoTmp KSASRR-YGIIQCDELVFKGLERTARHMVIPYMPMLVPPVKWTGYDKGGHLYLPSYVMR-

VvRpoTmp KKIGRR-FGVIECDPLVKKGLERTARHMVIPYVPMLVPPLKWTGYDKGAHLFLPSYVMR-

PotRpoTmp KKTSRK-YGVIQCDSLVLKGLEQTDRHIVIPYMPMLVPPLRWRGYDKGAHLFLPSYVMR-

AtRpoTmp MNSGRK-YGVIECDPLVRKGLEKSGRYAVMPYMPMLVPPLKWSGYDKGAYLFLTSYIMK-

CsRpoTmp KKSGRR-YGVIECDPLVRKGLEKTGWHMVMPYMPMLVPPVNWSGYDRGAYFFLPSYLMR-

SoRpoTmp KKTGRR-FGMIECDPLIIKGLEKTPRHIVIPYMPMLVPPEKWTGYDKGAYLVLRSFVMR-

AtRpoTm TKTSRR-YGCIECDPLVLKGLDKSARHMVIPYLPMLIPPQNWTGYDQGAHFFLPSYVMR-

BoRpoTm KKLSRK-YGCIECDPLIRKGLDKSARHMVIPYLPMLIPPQNWTGYDQGAHFFLPSYIMR-

NaRpoTp DN-SVKRYGIIECDPLVRKGLDATARHLVIPYMPMLIPPKKWTGYDKGGHLFLPSYVMR-

PpRpoTmp1 KNNMNRIYGVIECDPAVMSALDKSVQHMVMPYMPMLVKPRAWTGFYDGGYLHLKSTIMR-

PpRpoTmp2 -QNVNRIYGVIECDQLVLAEIDQSVKHMVMPYMPMLVKPLPWKGFNEGGYLYLKSSIMR-

PpRpoTm -GRSSTGFGVVECNPLVLEQIDKSVKYVIMPYMPMVSKPKHWKGFHDGGYLFLKSSIMR-

SmRpoTm KGGLLRSYGAIVCDPHLRGRIDDSAKFIVMPYMPMLIPPRAWKGYHNGAYLHLRSVIMR-

MspecRpoT RYGVISWHDAFFT--FMDDEAMIRATLSPVRYMPMVTPPRPWTRFNAGGYLRTESIVMRG

MipuRpoT RYGMVSWHPAFFD--YIEHEDMRHAQMSLVRYMPMVTPPRPWSRFDQGGYLCSETFVMRG

OlRpoT GYGMLHWHDSIYR--FINTETMTRAALVPVRHFPMVIPPRYWERYNKGGYLRADNLCMRG

OtRpoT GYGMLHWHDSIYR--FINTETMTRAALVPVKHFPMIVPPREWVRYNNGGYLRANTLCMRG

PotRpoTp1 ---THG-SRQQQVAVRSVPG--------KQMQKVFEALDTLGNTKWRVNRRLLDVVERIW

PotRpoTp2 ---THG-SRQQQDAVRSVPG--------KQMQKVFEALDTLGNTKWRVNGKVLDVVERIW

VvRpoTp ---THG-SRKQQDAVKSVPR--------RQLQKVFEALDTLGNTKWRINRRVLSVVESIW

NsRpoTp ---THG-SRRQQDAVRSVPT--------KQMQQVYEALDTLGSTKWRVNKRILSVVESIW

AtRpoTp ---THG-SKKQQDALKDISH--------KTAHRVFEALDTLGNTKWRVNRNILDVVERLW

SoRpoTp ---THG-SRKQQDAMRKTPP--------QQMQKVFEALNTLGHTKWRINRKVLGVVENLW

ZmRpoTp ---THG-VKDQKDAINSVPR--------KQLRKVFEALDILGSTKWRVNRRVHDVVETIW

SbRpoTp ---THG-VKDQKDAINSVPR--------KQLRKVFEALDILGSTKWRVNRRVHDVVETIW

OsRpoTp ---THG-VKDQKEAIKSVPR--------KQLRKVFEALDTLGSTKWRVNRRVHNAVETIW

HvRpoTp ---THG-VKDQKEAIKSVPR--------KQLRKVFEALDILGGTKWRVNRRVHDVVETIW

TaRpoTp ---THG-VKDQKEAIKSVPR--------KQLRKVFEALDILGGTKWRVNRRVHDVVETIW

SbRpoT1 ---THG-ARQQREAVKRAPR--------EQMQSVFEALNTLGSTKWRVNKRVLSIVDRIW

ZmRpoTm ---THG-ARQQRDAVKRAPR--------EQMQFVFEALNTLGSTKWRVNKRVLSIVDRIW

HvRpoTm ---THG-ARQQREAVKKAPK--------EQMQTIFEALDNLGSTKWRVNKRVLSIVDRIW

TaRpoTm ---THG-ARQQREAVKKAPK--------EQMQTIFEALDNLGSTKWRVNKEVLSIVDRIW

OsRpoTm ---THG-ARQQRDAVRRAPR--------EQMQSVFEALNTLGSTKWRVNKRVLSIVDRIW

NaRpoTm2 ---THG-SRQQREAVKRTPT--------QQLQSIFEALDTLGSTKWRINKRLLAVVERIW

NaRpoTm1 ---THG-ARQQREAVKSAPR--------KQLQSVFEALDTLGSTKWRVNKRVLAVVDRIW

VvRpoTm ---IHG-ARQQREAIKRAPR--------KALEPAFEALNTLGNTKWRINKRVLGVIDRIW

NsRpoTm ---THG-AKQQREAVKRVPK--------KQLEPVFQALDTLGNTKWRLNRKVLGIVDRIW

PotRpoTm1 ---IHG-SKQQRDAVKRASR--------NQLEPVFKALDTLGNTKWRINKRVLVVVDRIW

PotRpoTm2 ---IHG-AKQQRVAIKRASR--------NQLEPVFKALDTLGNTKWRVNKRILGVIDRIW

RcRpoTm ---THG-AKQQRNAVKRTPW--------KQLEPVYEALNTLGNTKWRINKKILTVVDRIW

CaRpoTm ---THG-VKQQRDAIKRVARD-------NNLCTVFEALDTLGNTKWRVNKKVLSIIDRIW

NsRpoTmp ---THG-ARQQREAVKRASR--------NQLQPVFEALDTLGNTKWRINKRVLSVVDRIW

VvRpoTmp ---THG-ARQQREAVKRAPR--------NQLEPVFEALNTLGNTKWRINKRLFDVVDRIW

PotRpoTmp ---IHG-AKQQREAVKRTPK--------KQLQLVFEALDTLGNTKWRVNKRVLSVVDRIW

AtRpoTmp ---THG-AKQQREALKSAPK--------GQLQPVFEALDTLGSTKWRVNKRVLTVVDRIW

CsRpoTmp ---IHG-AKQQREALKSTPR--------KQLQLVFEALDTLGNAKWRVNKRVLSIVDRIW

SoRpoTmp ---THG-SKHQRVAVRRASE--------EQLKSVFEALDTLGNTKWRVNKKVLSIVDRVW

AtRpoTm ---THG-AKQQRTVMKRTPK--------EQLEPVYEALDTLGNTKWKINKKVLSLVDRIW

BoRpoTm ---THG-AKQQRIAIKRTPK--------AQLEPVFQGLNTLGNTKWRINKKVLSLVDRIW

NaRpoTp ---THG-AKQQRVALKSIPK--------EQLKKVFEALDTLGSTKWRVNKRVLDVIDSLW

PpRpoTmp1 ---THG-AKELRDTIISTLR--------QDMIKIVQALDALGSTQWKINNVVLDVLEQMW

PpRpoTmp2 ---TQG-AKEQRMAVIDTPR--------KHMKVVVEALNVLGETGWRVNKRVLEVVEKLW

PpRpoTm ---THG-SKEQYDIFKNTPR--------ENMKKIFQALNVLGETGWRVNKPVLAVLEQIW

SmRpoTm ---THG-SKQQREAVRNTPR--------AQLQQVFRALDTLGAASWKINKEVFEVIEKLW

MspecRpoT HYTTHGPSKRQMLALHGEQSGAELDGRAAKFQPVLDALNVLGRTAWRINEDVLRVMEEVW

MipuRpoT NYTRLGPSWGQIMALLGEQKRSDSDGECASYQPVLDALNVLGKTPWVVNERTVDVMQKVW

OlRpoT KYSNEGPSRAQIAALEEKAREADASGEPVQYQPVLDALNALGQTAWQINTDVLPIVEEVW

OtRpoT KYSNEGPSRAQVQALEAKAREAELKNEPVLYQPVLDALNALGRTPWQVNTDVLPIVEEVW

PotRpoTp1 TSGGNIAGLVDREDIPIPEKPSS------------------DDLTEIQKWKWSVRKAKKI

PotRpoTp2 ASGGNNAGLVNRGDIPIPEKLSS------------------DDLTEIQKWKWSVRKAKKI

VvRpoTp ARGGNLGGLVDRENVPLPEKPST------------------EDLTEIQNWKWNARKAKKI

NsRpoTp AGGGNIAGLVDRKDVPIPELHS-------------------DDIMEVKKWKWRVRKSKKI

AtRpoTp ADGGNIAGLVNREDVPIPEKPSS------------------EDPEELQSWKWSARKANKI

SoRpoTp ASGGNIGGLVNCDDVPLPEKPLT------------------EDPAEIDTWKWSVRKAKKI

ZmRpoTp SQGGGIAGLVDKANIPLPERPES------------------EDPDEMQKWKWSLKKAKKT

SbRpoTp SRGGGIAGLVDKANIPLPERPES------------------EDPDEMQKWKWSLKKAKKT

OsRpoTp SRGGGIAGLVDKENIPLPERPET------------------EDPDEIQKWKWSLKKAKKA

HvRpoTp SRGGGIAGLVDKENIPLPERPET------------------EDPDEIQKWRWSVKKTKKT

TaRpoTp SRGGGIAGLVDKGNIPLPEQPET------------------EDPDEIQKWKWSVKKTKKA

SbRpoT1 SSGGRLADLVDRTDVPLPEKPDT------------------EDETLIKNWKWHLRSVKKE

ZmRpoTm SNGGRLADLVDRTDVPVPEKPDT------------------EDETLLKNWKWHLRAAKKK

HvRpoTm SSGGRLADLVDRADVSLPEKPDT------------------EDEAELKKWRWSMRSAKKE

TaRpoTm SSGGRLADLVDRADVSLPEKPDT------------------EDEAELKKWRWSMRSAEKE

OsRpoTm SSGGRLADLVDRTDVALPEKPDT------------------EDEDKLKKWRWTLRAAKKE

NaRpoTm2 ASGGNLAGLVDCEDAPLPEKPDT------------------EDEAVLRKWKWSLRNTKKE

NaRpoTm1 ASGGHLAGLVDCQDVPLPEKPDT------------------EDEAVLRKWKWSLRNARKE

VvRpoTm ASGGRLADLVDREDVPLPEEPDT------------------EDEAEIRKWKWKVRSVKKE

NsRpoTm ASGGRLADLVDREDVPLPEEPDA------------------EDEAQIRKWKWKVKGVKKE

PotRpoTm1 ASGGHLAGLVDREDAPLPEEPQT------------------EDEAEIRKWTWKVRSVKKE

PotRpoTm2 ASGGHLAGLVDREDVPLPEEPQT------------------EDEAEIRKWRWKVKSVKKE

RcRpoTm ANGGRLAGLIDREDEPLPEELET------------------EDENEIKKWKWKVKNVKKE

CaRpoTm ASGGCLADLVDREDMPLPGEPDT------------------DDAEEIKKWKWKVKHVKKD

NsRpoTmp AGGGRLADLVDRDDAPLPEEPDT------------------EDEALRTKWRWKVKSVKKE

VvRpoTmp AGGGCLADLVDRNDVPLPEKPDT------------------EDEAQLRKWKWKVKSVKKA

PotRpoTmp NNGGRLADLVDRSDVPLPEKPET------------------EDEALLKKWKWKVKSVKKE

AtRpoTmp SSGGCVADMVDRSDVPLPEKPDT------------------EDEGILKKWKWEVKSAKKV

CsRpoTmp NSGGRLADLVDRSDVPLPEKPDT------------------EDENILKKWKWKVKSANKE

SoRpoTmp ANGGRLADLVDRDDVPLPEKPDT------------------EDESELKRWKWKVRSLKKE

AtRpoTm ANGGRIGGLVDREDVPIPEEPER------------------EDQEKFKNWRWESKKAIKQ

BoRpoTm ANGGRVGGLVDRDDVPIPEEPDS------------------EDQEEIKQWKWKMKEANKE

NaRpoTp ASGRPIAGLVDHSDIALPEKPCT------------------EDEAHMRRWRWSVKKVKKE

PpRpoTmp1 KDGGRLADLVDAEDVPLPDKPES------------------DDFEEIRNWRRHTASAKRT

PpRpoTmp2 KAGGGIADLVEADDVPIPERPDT------------------SDKEVWHKWKVAVSQAKRT

PpRpoTm KEGGRLANLVDAEDVLVPAKPET------------------NNLDELKSWRREVGIVKRT

SmRpoTm SAGGGIGGLVDRKDVPLPPKPNT------------------EDEAEMRSWRKVFYKGKRT

MspecRpoT ATGGGRADVPPRENVPDPTWPANPYGLR--RSRGQLAATALPSRQDVAGFIHSLNRTKQR

MipuRpoT DDGGGIAGVPPRRTVDEPVWP------------------------DPGEYVNAKVRVKRE

OlRpoT ARGGGVAEVPLRAELQLPRWPGG----------------GLPGKGEVIDFLQSVRKTKKS

OtRpoT ARGGGIAEVPLRAELQLPRWPGGSYALRSDKNRFQLLASGLPGKGEVIEFLQSVRKTKKS

PotRpoTp1 NQERHSQRCDTELKLSVARKLKDEEGFYYPHNLDFRGRAYPMHPHLTHLSSDLCRGVLEF

PotRpoTp2 NQERHSQRCDTELKLSVARKLKDEEGFYYPHNLDFRGRAYPMHPHLTHLSSDLCRGVLEF

VvRpoTp NQERHSLRCDTEIKLSVARKMKDEEGFYYPHNLDFRGRAYPMHPHLNHLSSDLCRGVLEF

NsRpoTp NQELHSQRCDTELKLSVARKLKDEEGFYYPHNLDFRGRAYPMHPHLNHLSSDLCRGILEF

AtRpoTp NRERHSLRCDVELKLSVARKMKDEEGFYYPHNLDFRGRAYPMHPHLNHLSSDLCRGTLEF

SoRpoTp NRERHALRCDVELKISVAQKMKDEEGFYYPHNLDFRGRAYPMHPHLNHLSSDLCRGVLEF

ZmRpoTp NRELHAERCDTELKLSVARKMREEDGFYYPHNLDFRGRAYPMHPHLSHLGSDLCRGVLEY

SbRpoTp NRELHAERCDTELKLSVARKMREEDGFYYPHNLDFRGRAYPMHPHLSHLGSDLCRGVLEY

OsRpoTp NRELHAERCDTELKLSVARKMREEDGFYYPHNLDFRGRAYPMHAHLSHLGSDLCRGVLEY

HvRpoTp NRELHAERCDTELKLSVARKMREEDGFYYPHNLDFRGRAYPMHPHLSHLGSDLCRGVLEY

TaRpoTp NRELHAERCDTELKLSVARKMREEDGFYYPHNLDFRGRAYPMHPHLSHLGSDLCRGVLEY

SbRpoT1 NSERHSQRCDVELKLAVARKMKDEEGFYYPHNLDFRGRAYPMHPYLNHLGSDLCRGILEF

ZmRpoTm NSERHSQRCDVELKLAVARKMKDEEGFYYPHNLDFRGRAYPMHPYLNHLGSDLCRGVLEF

HvRpoTm NSERHSQRCDVELKLAVARKMKEEVGFYHPHNLDFRGRAYPMHPYLNHLGSDLCRGVLEF

TaRpoTm NSERHSQRCDVELKLAVARKMKEEAGFYYPHNLDFRGRAYPMHPYLNHLGSDLCRGVLEF

OsRpoTm NSERHSQRCDVELKLAVARKMKDEDGFYYPHNLDFRGRAYPMHPYLNHLGSDLCRGVLEF

NaRpoTm2 NSEKHSQRCDVELKLSVARKMKEEEGFYYPHNLDFRGRAYPMHPYLNHLGSDLCRGILEF

NaRpoTm1 NSERYSQRCDVELKLAVARKMKDEEGFYYPHNLDFRGRAYPMHPYLNHLGSDLCRGVLEF

VvRpoTm NSERHSQRCDIELKLAVARKMKDEDGFFYPHNLDFRGRAYPMHPYLNHLGSDLCRGILEF

NsRpoTm NCERHSQRCDIELKLAVARKMKDEDGFYYPHNLDFRGRAYPMHPYLNHLGSDLCRGILEF

PotRpoTm1 NSERHSQRCDIELKLAVARKMKDEEGFYYPHNLDFRGRAYPMHPYLNHLGSDVCRGILEF

PotRpoTm2 NSERHSQRCDVELKLAVARKMKDEEGFYYPHNVDFRGRAYPMHPYLNHLGSDVCRGILEF

RcRpoTm NSERHSQRCDIELKLAVARKMKDEDGFYYPHNLDFRGRAYPMHPHLNHLGSDMCRGILEF

CaRpoTm NAERHSQRCDTELKLAVARKMKDEEGFYYPHNLDFRGRAYPMHPHLNHLGSDLCRGILEF

NsRpoTmp NRERHSQRCDIELKLAVARKMKDEESFFYPHNVDFRGRAYPMHPHLNHLGSDICRGVLEF

VvRpoTmp NSERHSQRCDIELKLAVARKMKDEDGFFYPHNLDFRGRAYPMHPYLNHLGSDLCRGILEF

PotRpoTmp NRERHSRRCDTELKIAVARKMKNEEGFYYPHNLDFRGRAYPMHPYLNHLGSDLCRGILEF

AtRpoTmp NSERHSQRCDTELKLSVARKMKDEEAFYYPHNMDFRGRAYPMPPHLNHLGSDLCRGVLEF

CsRpoTmp NRERHSQRCDIELKLNVARKMKDEEGFYYPHNIDFRGRAYPMHPHLNHLGSDLCRGVLEF

SoRpoTmp NRERHSQRCDVELKLAVARKMKDEEGFFYPHNVDFRGRAYPMHPYLNHLGSDMCRGILEF

AtRpoTm NNERHSQRCDIELKLEVARKMKDEEGFYYPHNVDFRGRAYPIHPYLNHLGSDLCRGILEF

BoRpoTm NSERHSQRCDVELKLEVARKMKDEEGFYFPHNVDFRGRAYPMHPYLNHLGSDLCRGILEF

NaRpoTp NCERHAQRCDIELKLSVARKMRKEDGFYYPHNLDFRGRAYPMHPHLNHLGSDLCRGILEF

PpRpoTmp1 NSERHSVRCDTELKLAAARKLRDEEGFFYPHNLDFRGRAYPIHPHLNHLGSDMCRGILQF

PpRpoTmp2 NSERHSLRCDTELKLGVAKKLIDEEAFYYPHNLDFRGRAYPMHPHLNHLGNDLCRGLLIF

PpRpoTm NYERYSLRCDVELKLAVARKLVNEDAFYLPHNLDFRGRAYPMHPNLNHLGSDMCRGVLEF

SmRpoTm NSERNSQRCDLELKLAVARSLKDEECFYYPHNLDFRGRAYPMHAHLNHLGSDVCRGMLLF

MspecRpoT NRELHSQRCDFLIKLQVAKEMRDEDRIYFPHNIDFRGRAYTMHVHLNHLGSDICRGALVF

MipuRpoT NRELHSQRCDFLIKLKVAQEMRDEEKIFFPHNIDFRGRAYTMHAHLNHIGSDVCRGALLF

OlRpoT NMELHSQRCDFLIKLQVAREMKNEPNIYFPHNLDFRGRAYTMHVHLNHIGSDLCRGLLRF

OtRpoT NMELHSQRCDFLIKLQVAREMKNEPNIYFPHNLDFRGRAYTMHVHLNHIGSDLCRGLLRF

PotRpoTp1 EEGRPLGKSGLRWLKIHLANLYSGG-VEKLSHDGRLAFVENHLSEIFDSAKNPVNGK---

PotRpoTp2 AEGRPLGKSGLCWLKIHLANLYSGG-VEKLSHDGRLAFVENHLSEIFDSAENPVNGK---

VvRpoTp AEGRPLGKSGLRWLKIQLANLYAGG-VEKLSYDGRLAFVDNHLDDVFDSADNPLNGN---

NsRpoTp AEGRPLGKSGLRWLKIHLASLYAGG-IEKLCYDARLAFVENHIDDILDSANNPLNGN---

AtRpoTp AEGRPLGKSGLHWLKIHLANLYAGG-VEKLSHDARLAFVENHLDDIMDSAENPIHGK---

SoRpoTp ASGRPLGKTGLHWLKIHLANVYAGG-VDKLSYEARAAFVENHLEEVFDSATNPINGN---

ZmRpoTp AEGRPLGKSGLCWLKIHLANKYGGG-IEKLSHEGKLAFVENQLFDIFDSAANPVDGN---

SbRpoTp AEGRPLGKSGLCWLKIHLANKYGGG-VEKLSHEGKLAFVENQLLDIFDSAANPVDGN---

OsRpoTp AEGRPLGKSGLRWLKIHLANKYGGG-IEKLSHEDKVAFVENQLPDIFDSATNPVDGN---

HvRpoTp GEGRPLGKSGLRWLKIHLANKYGGG-IEKLTHESKLAFVEDRLPAIFDSAANPVDGN---

TaRpoTp AEGRPLGKSGLRWLKIHLANKYGGG-IEKLSHESKLTFVEDHLPDIFDSAANPVDGN---

SbRpoT1 AEGRPLGKSGLHWLKIHLANLYAGG-VDKLSYDGRVAFTENHLEEIFDSADRPLEGK---

ZmRpoTm AEGRPLGKSGLRWLKIHLANLYAAG-VDKLSYDGRIAFAENHLEEIFDSADRPLEGR---

HvRpoTm SEGRPLGESGLRWLKIHLANLYGGG-VDKLSYDGRIAFTENHLEDIFDSANRPLEGK---

TaRpoTm SEGRPLGESGLRWLKIHLANLYGGG-VDKLSYDGRIAFTENHLDDIFDSANRPLEGK---

OsRpoTm AEGRPLGKSGLRWLKIHLANLYAGG-VDKLSYDGRIAFTENHLEDIFDSADRPLEGK---

NaRpoTm2 AEGRPLGKSGLRWLKIHLANLYAGG-VDMLSYDGRLAFVENHLDDIFDSADKPLEGS---

NaRpoTm1 AEGRPLGKSGLHWLKIHVANLYAGG-IDKLSYDGRLAFVENHLDDIFDSSDRPLEGS---

VvRpoTm AEGRPLGKSGLRWLKIHLANVYAGG-VDKLSYEGRVAFTENHLEDIFDSADRPLEGR---

NsRpoTm AEGRPLGKSGLRWLKIHLANVYGGG-VDKLSYEGRVAFSENHVEDIFDSAERPLEGK---

PotRpoTm1 AEGRPLGKSGLRWLKIHLANLYAGG-VDKLSYDGRISFTENHLDDIFDSADQPLEGR---

PotRpoTm2 AEGRPLGKSGLRWLKIHLANLYAGG-VDKLSYDGRISFTENHLDDIFDSADRPLEGQ---

RcRpoTm AEGRPLGKSGFRWLKIHLANVYAGG-VDKLSYEGRVAFTENHLDYIFDSADRPLEGR---

CaRpoTm AEGRPLGKSGLNWLKVHVANLYAGG-VDKLSYEGRVAFTENRLDDVFDSADRPLEGR---

NsRpoTmp AEGRPLGESGLRWLKIHLANLFAGG-VEKLSLEGRIGFTENHMDDIFDSSDKPLEGR---

VvRpoTmp AEGRPLGSSGLHWLKIHLANLFAGG-VDKLSHEGRITFTENHLDDIFDSADRPLEGK---

PotRpoTmp AEGRPLGKSGLRWLKIHLANLFACG-VDKLSHEGRIAFTENHLDDIFDSADKPLEGK---

AtRpoTmp AEGRPMGISGLRWLKIHLANLYAGG-VDKLSLDGRLAFTENHLDDIFDSADRPLEGS---

CsRpoTmp AEGKPLGSSGLRWLKIHLANLYAGG-VDKLSFEGRLAFTENHLDDIFNSADRPLEGR---

SoRpoTmp AEGRAVGKSGLRWLKIHVANLYAGG-IDKLSLEGRIAFTENHLDDIFDSVDKPLEGR---

AtRpoTm CEGKPLGKSGLRWLKIHIANLYAGG-VDKLAYEDRIAFTESHLEDIFDSSDRPLEGK---

BoRpoTm CEGKPLGESGLRWLKIHIANLYGGG-VDKFAYKDRVAFAESHLEDIFDSSDRPLEGK---

NaRpoTp AEGKPLGKTGLRWLKIHLANLYGGG-VDKLSFDGRLTFVENHLADIFDSAERPIEGR---

PpRpoTmp1 AEGRALGPTGLRWLKIHLANLYGGK-VGKMSFDARVAWVDEVMEKVFDSADRPLDGS---

PpRpoTmp2 ADGKPLGPSGLRWIKIQLANLYGGS-VGKMSFDDRAAFAEDRMEEILDSAERPLDGS---

PpRpoTm AKGRPLGETGLRWLKIHLANLYGGS-ISKLSFDARVAHVDTHMDDVFDSAENPMNGN---

SmRpoTm AKGRPLGPTGLRWLKIHIANVFANG-ADKLPFDGRVAFAESNLEHVVASANQPLKN----

MspecRpoT ADARPLGNDGLDWLYIQAANLYAGG-VDKLPMDERRQWIEDRVHLLEQSARDPLGPDGG-

MipuRpoT QEARPLGENGLDWLYVQAANLYAAGGVDKLPLDERREWMKTRLDRIKASARDPLCADDAN

OlRpoT NEKKPLGERGLRWMHIQCATLFGNG-ADKLPMDERVQFIKDRIEDVRASAQDPLAKDAW-

OtRpoT SEKKPLGERGLRWMYIQCATLFGNG-ADKLPMDERVQFIVDQIEQVRASAQDPLAKGAW-

PotRpoTp1 -RWWLKAEDPFQCLAACINLSEALNSASPHTVISHLPIHQDGSCNGLQHYAALGRDTLEA

PotRpoTp2 -RWWLTAEDPFQCLAACINLSEALKSASPHTVISHLPIHQDGSCNGLQHYAALGRDSQEA

VvRpoTp -RWWLTAEDPFQCLAACINLSEALRSSSPHTVISHLPIHQDGSCNGLQHYAALGRNSLEA

NsRpoTp -RWWLNAEDPFQCLAACINLSEALKSSSPHTVFSHLPIHQDGSCNGLQHYAALGRDSMEA

AtRpoTp -RWWLKAEDPFQCLAACVILTQALKSPSPYSVISHLPIHQDGSCNGLQHYAALGRDSFEA

SoRpoTp -RWWLSAEDPFQCLAACIDLTGALNSSSPHSFISYLPIHQDGSCNGLQHYAALGRDSKEA

ZmRpoTp -CWWTNAEDPFQCLAACMDLSDALRSPSPYHAVSHLPIHQDGSCNGLQHYAALGRDYMGA

SbRpoTp -CWWTNAEDPFQCLAACMDLSDALNSPSPYRTVSHLPIHQDGSCNGLQHYAALGRDYMGA

OsRpoTp -CWWMNAEDPFQCLAACMDLSDALKSSSPQCAVSHLPIHQDGSCNGLQHYAALGRDYMGA

HvRpoTp -CWWINAEDPFQCLAACIDLSDALKSSSPHAAVSHLPIHQDGSCNGLQHYAALGRDYMGA

TaRpoTp -CWWINAEDPFQCLAACMDLSNALESSSPHGAVSHLPIHQDGSCNGLQHYAALGRDYMGA

SbRpoT1 -RWWLGAEDPFQCLAVCMNLTEALRSSSPETTISHIPVHQDGSCNGLQHYAALGRDKLGA

ZmRpoTm -RWRLGAEDPFQCLAVCMNLTEALRSSSPETTISHIPVHQDGSCNGLQHYAALGKDKLGA

HvRpoTm -RWWLEAEDPFQCLAVCMDLNEALRSSSPETVISHIPVHQDGSCNGLQHYAALGRDKLGA

TaRpoTm -RWWLEAEDPFQCLAVCMDLNEALRSPSPETVISHIPVHQDGSCNGLQHYAALGRDKLGA

OsRpoTm -RWWLGAEDPFQCLAVCINLTEALRSPSPETMISHIPVHQDGSCNGLQHYAALGRDKLGA

NaRpoTm2 -RWWLGAEDPFQCLAACINLSEALRSSSPDTTISHVPVHQDGSCNGLQHYAALGRDKLGA

NaRpoTm1 -RWWLGAEDPFQCLAACINLSDALRSSSPETTISHVPIHQDGSCNGLQHYAALGRDKLGA

VvRpoTm -RWWLSAEDPFQCLATCINLSEALRSSSPETTISHMPVHQDGSCNGLQHYAALGRDKLGA

NsRpoTm -RWWLGAEDPFQCLATCINIAEALRSPSPETAISYMPIHQDGSCNGLQHYAALGRDTLGA

PotRpoTm1 -RWWLGAEDPFQCLAACINLSEALRSPSPETATSHTPVHQDGSCNGLQHYAALGRDKLGA

PotRpoTm2 -RWWLGAEDPFQCLAVCINLSEALRSPSPETAISHTPVHQDGSCNGLQHYAALGRDKLGA

RcRpoTm -RWWLGAEDPFQCLATCFNLAEAFRSSSPETTISHMPIHQDGSCNGLQHYAALGRDKLGA

CaRpoTm -RWWLTAEDPFQCLAACINLTDALRSSSPEDYLSHIPIHQDGSCNGLQHYAALGRDKLGA

NsRpoTmp -RWWLNAEDPFQCLAVCINLSEAVRSSSPETSVSHIPVHQDGSCNGLQHYAALGRDKLGA

VvRpoTmp -RWWLNAEDPFQFLAACINLSEALRSSSPETTISHIPVHQDGSCNGLQHYAALGRDQLGA

PotRpoTmp -RWWLHAEDPFQCLAVCINLTEALRSSSPETCLSHIPIHQDGSCNGLQHYAALGRDKLGA

AtRpoTmp -RWWLQAEDPFQCLAVCISLTEALRSPSPETVLSHIPIHQDGSCNGLQHYAALGRDTLGA

CsRpoTmp -RWWLQAEDPFQCLAVCINITEALRSPCPETFVSHIPIHQDGSCNGLQHYAALGRDTLGA

SoRpoTmp -CWWLKAEDPFQCLAVCIELAEALRSTRPEAFKSHIPVHQDGSCNGLQHYAALGRDELGA

AtRpoTm -RWWLNAEDPFQCLAACINLSEALRSPFPEAAISHIPIHQDGSCNGLQHYAALGRDKLGA

BoRpoTm -RWWLNAEDPFQCLAACMNLSEALRSPFPEAAVSHIPIHQDGSCNGLQHYAALGRDQIGA

NaRpoTp -QWWLNAEDPFQCLAACIDLSEALKSSSPEDAISHIPVHLDGSCNGLQHYAALGRDRLGA

PpRpoTmp1 -RWWLDAEDPFQFLATCLDIRNAIKSGNPETYVSHLPVHQDGSCNGLQHYAALGRDRIGA

PpRpoTmp2 -RLWLKAEDPFQFLAACIDLRDALASGNPETFVSHLPVHQDGSCNGLQHYAALGRDRIGA

PpRpoTm -RWWLKAEDPFQFLAACIDIRNAVKSGNPKTYNSFLPVHQDGSCNGLQHYAALGRDRIGA

SmRpoTm -RWWLKAEDPFQCLAACIDLRNAMHSPNPEYYISHLPVHQDGSCNGLQHYAALGRDKTGA

MspecRpoT --FWLDAEDPWQCLACCFEIERARASGDPPAYMCRLPVHQDGSCNGLQHYAALGRDEAGG

MipuRpoT CAFWLEAENPWQCLATFIEIAAAVDSGDPASYACRLPVHQDGSCNGLQHYAALGRDIGGG

OlRpoT ---WQEAEEPWQCLATCIELDKALELSDPTQFMSNLPVHQDGSCNGLQHYAALGRDLHGG

OtRpoT ---WQDAEEPWQCLATCIELDKALELEDPTQFLSNLPVHQDGSCNGLQHYAALGRDLHGG

PotRpoTp1 AAVNLVAAEKPSDVYSEIAVRVHEIIRRDSN----KDPATNPHALLAKIL-VDQVDRKLV

PotRpoTp2 AAVNLVAAEKPSDVYSEIATRVHEIIRRDSN----KDPATNPHALLAKVL-IDEVDRKLV

VvRpoTp SAVNLVAGEKPADVYSEIAARVHEIMKRDSN----KDPATDPKALLAKVL-IGQVDRKLV

NsRpoTp AAVNLVAGDKPADVYTEIALRVDHIIRGDSI----KDPATDPNALLAKLL-IDQVDRKLV

AtRpoTp AAVNLVAGEKPADVYSEISRRVHEIMKKDSS----KDPESNPTAALAKIL-ITQVDRKLV

SoRpoTp AAVNLVAGEEPADVYSEIAVRVREIMEKDSR----KDPKTDPHAVLAKVL-VDQVDRKLV

ZmRpoTp VAVNLVPGEKPADIYSEIASRVLNVVCEDSM----KDPATNPTASLARAL-VDQVDRKLV

SbRpoTp VAVNLVPGEKPADIYSEIAARVLNVVREDSM----KDPATNPTASLDMGIGSLQVDRKLV

OsRpoTp AAVNLVPGDKPADIYSEIAARVLDVVREDSM----EDPATNPTASLARVL-VDQVDRKLV

HvRpoTp AAVNLVPGEKPADIYSEIAARVLNVVREDSM----KDPATDPSVPLARVL-VDQVDRKLV

TaRpoTp AAVNLVPGEKPADIYSEIAARVLDVVREDSM----KDPATDPSVPLAKVL-VDEVDRKLV

SbRpoT1 IAVNLVAGEKPADVYTGIATRVLEIMRMDAQ----KDPSIDPDVARARLI-VDQVDRKLV

ZmRpoTm IAVNLVAGEKPADVYTGIANRVMEIMRMDAQ----KDPSVEPDAARARLI-VDQVDRKLV

HvRpoTm VAVNLVSGEKPADVYSGIAARVVEIMKRDAQ----KDPAKDADAARARLL-VDQVDRRLV

TaRpoTm VAVNLVSGEKPADVYSGIAARVVEIMKRDAQ----KDPAKDADAARARLL-VDQVDRKLV

OsRpoTm IAVNLVAGEKPADVYTGIATRVVEIMKNDAL----KDPATDPDAARARLL-LDQVDRKLV

NaRpoTm2 ASVNLVAGEKPADVYSGIASGVLEIMRRDSQ----KDPANNPSAVRARLL-IDQVDRKLV

NaRpoTm1 AAVNLVAGEKPADVYSGIAERVFDIMRRDSQ----KDPTNNPSAARARLL-IDQVDRKLV

VvRpoTm AAVNLVAGEKPADVYSGIAARVLDIMQRDAQ----KDPATEPNALRARLL-ISQVDRKLV

NsRpoTm AAVNLVAGDKPADVYSGIAARVLDIMKRDAA----KDPANDPNVMRARLL-INQVDRKLV

PotRpoTm1 AAVNLVGGEKPADVYSGIATRVLDIMQRDAE----KDPAINPNSVHAKLL-VNQVDRKLV

PotRpoTm2 AAVNLVGGGKPADVYSGIAARVLDIMRRDAE----NDPAINPNSVHAKLL-INQVDRKLV

RcRpoTm AAVNLVSGEKPADVYSGIAARVLDIMRTDAE----KDPGTNPNALHAKLL-INEVDRKLV

CaRpoTm EAVNLVAGGKPADVYSGIAARVFEIMRGDAE----KDPSIEPNAFHAKLL-LNQVDRKLV

NsRpoTmp AAVNLVAGEKPADVYSGIAARVLDIMKRDAQ----RDPAEFPDAVRARVL-VNQVDRKLV

VvRpoTmp TAVNLVAGEKPADVYSGIAARVLDIMKRDAE----KDPAIFPDALRARIL-INQVDRKLV

PotRpoTmp AAVNLVAGEKPTDVYSGIAARVLDIMRSDAQ----EDPEVFPDALRARRL-ISQVDRKLV

AtRpoTmp EAVNLVAGEKPADVYSGIATRVLDIMRRDAD----RDPEVFPEALRARKL-LNQVDRKLV

CsRpoTmp AAVNLVAGEKPADVYSGIAARVLDIMRRDAE----QDPEVFPDALRARNL-VNQVDRKLV

SoRpoTmp TAVNLVAGEKPADVYSGIASRVLSIMEADAK----KDPTDFPDTVLARKL-INQVDRKLV

AtRpoTm DAVNLVTGEKPADVYTEIAARVLKIMQQDAE----EDPETFPNATYAKLM-LDQVDRKLV

BoRpoTm AAVNLFTGEKPADVYADIAARVLNIMRQDAE----EDPETFPNATYAKLM-LDQVDRKLV

NaRpoTp ASVNLVSGEKPTDVYSGIAARVMEIVVRDSK----KDPAVHPTSLLARIL-IDQVDRKLV

PpRpoTmp1 ESVNLVAGDKPADVYSGIAARVREIMERDAQ----KDPKTSRHAANAKLL-LPEIDRKLV

PpRpoTmp2 ESVNLIAGDKPADVYSGIAERVKIIMEKDAL----KNPLTSRNAASARLL-QGQIDRKLV

PpRpoTm GTVNLLAGDVPADVYSAIADRVHRTIEKAAL----KNPEASKHAAIARVL-LGQIDRKLV

SmRpoTm RAVNLIGADYPADVYSGIAARVRTLVEEDAR----KDP----AAVYAKLL-VGHVDRKLV

MspecRpoT EQVNLMPRDRPGDVYTGIANVLKRIVAEDAK-----NPEDPETQALAIAL-APHVDRKLV

MipuRpoT EQVNLTPRDSPGDVYTGVANVLKRMVAEDAA-----DASDPETAATAAAL-APHIDRKLV

OlRpoT EAVNLVPADRGADVYTGIANVLKRIVAEDIKLIDSEDEEDVNNAKLAMSL-AQHIDRKLV

OtRpoT EAVNLVPADRGADVYTGIANVLKRIVADDIKLIDSDNEEDVANAKLAMAL-AHHIDRKLV

PotRpoTp1 KQTVMTSVYGVTYVGAREQIKRRLEEKGHITDD-RLLFSAACYTAKVTLTALGELFQAAR

PotRpoTp2 KQTVMTSVYGVTYVGAREQIKRRLEEKGHITDD-RVLFSAACYAAKVTLTALGELFQAAR

VvRpoTp KQTVMTSVYGVTYIGAREQIKRRLAEKGLITDE-RLLFTAACYAAKVTLAALGEIFQAAR

NsRpoTp KQTVMTSVYGVTYVGAREQIKRRLEEKGLIDDD-RLLFTASCYAAKVTLAALGELFQAAR

AtRpoTp KQTVMTSVYGVTYVGAREQIKRRLEEKGVITDE-RMLFAAACYSAKVTLAALGEIFEAAR

SoRpoTp KQTVMTSVYGVTFVGAREQIKRRLEEKGVITDD-RLLFSAACYSAKVTMSALGQLFQAAR

ZmRpoTp KQTVMTSVYGVTYIGARQQITKRLQEKGLITDD-KLLYDVSCYATRVTLDALGQMFQSAR

SbRpoTp KQTVMTSVYGVTYIGARQQITKRLQEKGLIADD-KLLYDVSCYATRVTLDALGQMFQSAR

OsRpoTp KQTVMTSVYGVTYIGARQQITKRLQEKGLITDD-KLLYEVSCYATRVTLDALGQMFQSAR

HvRpoTp KQTVMTSVYGVTFIGARQQIMKRLQEKGHITDE-KLLYDVSCYAARVTLDALGQMFQSAR

TaRpoTp KQTVMTSVYGVTFIGARQQIMKRLQEKGHITDD-KLLYDVSCYATRVTLDALGQMFQSAR

SbRpoT1 KQTVMTSVYGVTYVGARDQIKRRLKERGVIPDE-AELFGASCYAAKVTLTALGEMFQAAR

ZmRpoTm KQTVMTSVYGVTYIGAREQIRRRLKERGVIPND-SELFGASCYAAKVTLTALGEMFQAAR

HvRpoTm KQTVMTSVYGVTYVGAREQIKRRLKERGVIADD-SELFGASCYAAKVTLTALGEMFEAAR

TaRpoTm KQTVMTSVYGVTYVGAREQIKRRLKERGVIADD-SELFGASCYAAKVTLTALGEMFEAAR

OsRpoTm KQTVMTSVYGVTYVGAREQIKRRLKERDMICDD-SELFSASCYAAKVTLTALGEMFQAAR

NaRpoTm2 KQTVMTSVYGVTYIGARDQIKKRLKERDVIPED--QVFGAACYAAKTTLAALEEMFQAAR

NaRpoTm1 KQTVMTSVYGVTYIGARDQIKRRLKERDAIPDD--QVFGAACYAAKTTLTALEEMFQAAR

VvRpoTm KQTVMTSVYGVTYIGARDQIKRRLKERCAIADE-GEVFSASCYAAKTTLTALGEMFEAAR

NsRpoTm KQTVMTSVYGVTYIGARDQIKRRLKERGVIEDD-NELFAAACYAAKTTLTALGEMFEAAR

PotRpoTm1 KQTVMTSVYGVTYIGARDQIKRRLKERCIIADD-PQLYSAACYAAKTTLMALEEMFEGAR

PotRpoTm2 KQTVMTSVYGVTYIGARDQIKRRLKERCNIADD-PQLYSAACYAAKTTLTALEEMFEGAR

RcRpoTm KQTVMTSVYGVTYIGARDQIKRRLKERDAVADD-PSLYAASCYAAKTTLMALEEMFEGAR

CaRpoTm KQTVMTSVYGVTYIGVRDQIKRRLKERDAIVDD-NDLFAASCYAAKTTMTALEEMFEAAR

NsRpoTmp KQTVMTSVYGVTYIGARDQIKRRLKERGAIADD-SELFGAACYAAKVTLTALGEMFEAAR

VvRpoTmp KQTVMTSVYGVTYIGARDQIKRRLKERNAIADD-VELFGAACYAAKITLTALGEMFQAAR

PotRpoTmp KQTVMTSVYGVTYIGARDQIERRLKER-GLTDN-SEIFGCSCYAAKVTLTALGEMFEAAR

AtRpoTmp KQTVMTSVYGVTYIGARDQIKRRLKERSDFGDE-KEVFGAACYAAKVTLAAIDEMFQAAR

CsRpoTmp KQTVMTSVYGVTYIGARDQIKRRLKERSIFTDD-TELFGAACYAAKVTLTALGEMFQAAR

SoRpoTmp KQTVMTSVYGVTYIGAREQIKRRLKERAAFSDD-TEIYGAACYVARITLTALGEMFQAAR

AtRpoTm KQTVMTSVYGVTYSGARDQIKKRLKERGTFEDD-SLTFHASCYAAKITLKALEEMFEAAR

BoRpoTm KQTVMTSVYGVTYSGARDQIKKRLKERGAFADD-SQNFHASCYAARVTLKALEEMFEAAR

NaRpoTp KQTVMTSVYGVTYIGARDQIKRRLKERDFITDD-KLLFSAACYAAKVTLSALEEMFQAAR

PpRpoTmp1 KQTVMTSVYGVTFVGARMQIFNRLKERGTIQEQ-TEMYRAACYAAKTTLNALGEMFKEAR

PpRpoTmp2 KQTVMTSVYGVTFIGARMQILNRLKERSPIAVDPVDTYRAACYAAKVTLDALGEMFKEAR

PpRpoTm KQTIMTSVYGVTFVGARIQILNRLKERGIIQDG-GELFKASVYAAKVTLDALGEGFREAR

SmRpoTm KQTVMTSVYGVTYVGARNQITNRLRDKGFILDE-PTLYRTGCYAAKVTLNALGEMFGEAR

MspecRpoT KQTVMTSVYGVTHIGARQQIQNRLKERGAVED-ENLRYKMANYAARRTLDALANLFVNAR

MipuRpoT KQTVMTSVYGVTHVGARQQIQSRLRERGAIED-ESLRYRTANYASRRTLDALSNLFVNAR

OlRpoT KQTVMTSVYGVTFIGARAQIYSRLREREAMEDNELLRYRVSNYAAKRTLDALNNMFSNAR

OtRpoT KQTVMTSVYGVTFIGARAQIYSRLREREVMEDNELLRYRVSTYAARRTLDALNNMFSNAR

PotRpoTp1 DIMSWLGDCAKIIASEDQPVQWTTPLGLPVVQPYYKTERHLIKTSLQILALQREGSS--V

PotRpoTp2 DIMGWLGDCAKVIASEDQPVRWTTPLGLPVVQPYYKTQRHLIRTSLQVLALQREGSS--V

VvRpoTp DIMAWLGECAKVIASENQPVQWTTPLGLPVIQPYCKSERHLIRTSLQVLALQREGSV--V

NsRpoTp GTMTWLGDCAKVIASENQPVRWTTPLGLPVVQPYFKTQRHVIRTSLQVLALQREGDT--V

AtRpoTp AIMSWLGDCAKIIASDNHPVRWITPLGLPVVQPYCRSERHLIRTSLQVLALQREGNT--V

SoRpoTp GIMDWLGDCAKVIASENQPVRWTTPLGLPVVQPYCKSKRHLIKTSLQVLALQQEGEG--I

ZmRpoTp GIMAWLGDCAKMIASKNQPVKWTSPVGLPVVQPYKKYKNYMIRTSLQCLALRREGDA--I

SbRpoTp GIMAWLGDCAKMIASKNQPVKWTSPVGLPVVQPYKKYKNYMIRTSLQCLALRREGDA--I

OsRpoTp GIMAWLGDCAKMIASENHPVKWTSPVGLPVVQPYKKYKNYMIRTSLQCLALRREGDA--I

HvRpoTp GIMAWLGDCAKMIASKNQPVRWTSPVGLPVVQPYKKYKNYMIRTSLQCLALRREGDA--I

TaRpoTp AIMAWLGDCAKMIASKNQPVRWTSPVGLPVVQPYKKYKNYMIRTSLQCLALRREGDA--I

SbRpoT1 GIMNWLGDCAKVIACENEPVRWTTPLGLPVVQPYRKLGRHLIKTSLQVLTLQRETDK--V

ZmRpoTm SIMNWLGDCAKVIACENEPVRWTTPLGLPVVQPYRKLGRHLIKTSLQVLTLQRETDK--V

HvRpoTm SIMNWLGDCAKVIACENEPVRWKTPLGLPVVQPYRKLGRHLIKTSLQVLTLQRETDK--V

TaRpoTm SIMTWLGDCAKVIACENEPVRWTTPLGLPVVQPYRKLGRHLIKTSLQVLTLQRETDK--V

OsRpoTm SIMNWLGDCAKVIACENEPVRWTTPLGLPVVQPYRKLGRHLIKTSLQVLTLQRETDK--V

NaRpoTm2 GIMSWLGDCAKVIASENESVRWTTPLGLPVVQPYKKQGRCLV------LALQKETDK--V

NaRpoTm1 AIMSWLGDCAKVIASENEPVRWTTPLGLPVVQPYKKKGRCLVKTSLQVLALKRETDK--V

VvRpoTm SIMSWLGDCAKVIASENHPVRWNTPLGLPVVQPYRKLGRHLIKTSLQVLSLQRETDK--V

NsRpoTm SIMSWLGDCAKIIAMENHPVRWTTPLGLPVVQPYRKLGRHLIKTSLQILTLQRETDK--V

PotRpoTm1 GIMAWLGECAKVIASENQPVRWTTPLGLPVVQPYRQLGRHLIKTSLQVLTLKRETDK--V

PotRpoTm2 GIMAWLGECAKVIASENQPVQWTTPFGLPVVQPYRQLGRQLIKTSLQVLTLQRETDK--V

RcRpoTm SIMAWLGECAKVIASENQPVQWTTPLGLPVVQPYRQLGRHLIKTSLQMLALQKETDK--I

CaRpoTm SIMGWLGDCAKIIASENQPARWSTPLGLPVVQPYRNHGRRLVKTSLQVLTLRCDTDK--V

NsRpoTmp SIMTWLGECAKIIASENEPVRWTTPLGLPVVQPYRKIGRHLIKTSLQILTLQRETEK--V

VvRpoTmp SIMSWLGDCAKIIASENQPVRWTTPLGLPVVQPYRKLGRHLIKTSLQVLTLQRETET--I

PotRpoTmp SIMNWLGECAKIIASENEPVRWTTPLGLPVVQPYRKLGKHVIKTSLQFLTLQKETDK--V

AtRpoTmp AIMRWFGECAKIIASENETVRWTTPLGLPVVQPYHQMGTKLVKTSLQTLSLQHETDQ--V

CsRpoTmp DIMSWLGECAKIIASQNQPVRWTTPLGLPVVQPYRKMGRHVIKTSLQVLSLQCETDK--V

SoRpoTmp SIMSWLGDCAKIIASENQPVRWITPLGLPVVQPYCKKGRHLIKTSLQMLTLEKETEK--I

AtRpoTm AIKSWFGDCAKIIASENNAVCWTTPLGLPVVQPYRKPGRHLVKTTLQVLTLSRETDK--V

BoRpoTm AIMSWFGECAKIIASQNKAVCWTTPLGLPVVQPYRKNERHLVKTSLQTLSLQRETDK--V

NaRpoTp SIMSWLGDCAKVIASENEPVRWTTPLGLPVVQPYWKQSRHLVRTSLQVLALQRESNK--V

PpRpoTmp1 CIMSWLGDCAKIIASNGETVKWTTPLGLPVVQPYRKPGRHLVKTSLQVLALRNLDADQPV

PpRpoTmp2 CIMSWLGDCAKIIAAAGHTVRWTSPLGLPIVQPYRKHSRHLVKTSLQVLALRNTDDNHPV

PpRpoTm CIMNWLSECAQIIAHSGNSVKWTTPLGLEVVQPYRNPSRHLVKTALQDLHIRSVDVDSPV

SmRpoTm LIMNWLGQCAKVIANDGDSVRWITPLGLPVVQPYRRPGRHVVKTCLQCLILRTDTDQ-PV

MspecRpoT DVMAWLAECARVVCSQGRAVEWTTPLGLPVVQPYRVKSQRMIRTLVQSFTLQFDNDENSV

MipuRpoT EVMSWLGECARVVSKGGKPVQWTTPMGLPVVQPYKAPSRKVVRTIVQSFVLQYDSEGSAI

OlRpoT DVMGWLTTCATIATSAGEPVRWTTPLGLPVVQPYHSQRTKRVRTILQSFSLKVHDEQQPV

OtRpoT DVMGWLTDCAAIATATGEPVRWTTPLGLPVVQPYHRKSTKRVRTILQSFTIKTDDDKQPV

PotRpoTp1 QVRKQRTAFPPNFVHSLDGSHMMMTAVACRDAGLCFAGVHDSFWTHATDVDLMNRILREK

PotRpoTp2 QVRKQRTAFPPNFVHSLDGSHMMMTAVACRDADLRFAGVHDSFWTHARDADLMNRILREK

VvRpoTp VIRKQRTAFPPNFVHSLDGSHMMMTAVACRDAGLCFAGVHDSFWTHACDVDKMNQILREK

NsRpoTp EVRKQRTAFPPNFVHSLDGSHMMMTAVACRDAGLQFAGVHDSFWTHACDVDQMNRILREK

AtRpoTp DVRKQRTAFPPNFVHSLDGTHMMMTAVACREAGLNFAGVHDSYWTHACDVDTMNRILREK

SoRpoTp EVKKQRTAFPPNYVHSLDGTHMMMTAVACRDAGLQFAGVHDSFWTHACDVEKMNQILRVK

ZmRpoTp AIQRQKAAFPPNFVHSLDSSHMMMTAIACKESGLHFAGVHDSFWVHACDVDQMNQILRQQ

SbRpoTp AIQRQKAAFPPNFVHSLDSSHMMMTAIACKEAGLHFAGVHDSFWVHACDVDQMNQILRQQ

OsRpoTp ALQRQKAAFPPNFVHSLDSSHMMMTAIACKKAGLHFAGVHDSFWVHACDVDKMNQILREQ

HvRpoTp ATQRQKAAFPPNFVHSLDSSHMMMTAITCKEAGLHFAGVHDSFWVHACDVDKMNQILREQ

TaRpoTp ATQRQKAAFPPNFVHSLDSSHMMMTAITCKEAGLHFAGVHDSFWVHACDVDKMNQILREQ

SbRpoT1 MVKRQRTAFPPNFVHSLDGSHMMMTAVACKRQGLNFAGVHDSYWTHACDVDTMNKILREK

ZmRpoTm MVKRQKTAFPPNFVHSLDGSHMMMTAVACKRQGLNFAGVHDSYWTHASDVDTMNKILREK

HvRpoTm MVKRQRTAFPPNFVHSLDGSHMMMTAVACKKQGLYFAGVHDSYWTHACDVDTMNKILREK

TaRpoTm MVKRQRTAFPPNFVHSLDGSHMMMTAVACKKQGLYFAGVHDSYWTHACDVDTMNKILREK

OsRpoTm MVKRQRTAFPPNFVHSLDGSHMMMTAVACKRQGLNFAGVHDSYWTHACDVDTMNKILREK

NaRpoTm2 MIKRQRTAFPPNFVHSLDGSHMMMTAIACKKIGLNFAGVHDSYWTHACDVDEMNRILRKK

NaRpoTm1 MIKRQRTAFPPNFVHSLDGSHMMMTAVACKKAGLKFAGVHDSYWTHACDVDEINRILREK

VvRpoTm MVKRQRTAFPPNFVHSLDGSHMMMTAVACQKAGLNFAGVHDSYWTHACDVDEMNRILREK

NsRpoTm MVKRQRTAFPPNFVHSLDGSHMMMTAIACKESGLSFAGVHDSYWTHASDVDQMNKILREK

PotRpoTm1 MVKRQRTAFPPNFVHSLDGSHMMMTAVACKEAGLNFAGVHDSYWTHACDVDEMNRILREK

PotRpoTm2 MVKRQRTAFPPNFVHSLDGSHMMMTAVACKEAGLNFAGVHDSYWTHACDVDEMNRILREK

RcRpoTm MVKRQRTAFPPNFVHSLDGSHMMMTAVACKHAGLNFAGVHDSYWTHACDVDEMNRILREN

CaRpoTm MAKRQRTAFPPNFVHSLDGSHMMMTAIACKKAGMNFAGVHDSYWTHACDVDKMNQILREK

NsRpoTmp MVKRQRTAFPPNFIHSLDGSHMMMTAVACRRAGLNFAGVHDSYWTHACDVDKLNRILREK

VvRpoTmp MVKRQRTAFPPNFVHSLDGSHMMMTAIACKKAGLNFAGVHDSYWTHACDVDEMNRLLREK

PotRpoTmp MVKRQRTAFPPNFVHSLDGSHMMMTAVACKRAGLKFAGVHDSYWTHACDVDEMNRILREK

AtRpoTmp IVRRQRTAFPPNFIHSLDGSHMMMTAVACKRAGVCFAGVHDSFWTHACDVDKLNIILREK

CsRpoTmp MVKRQRTAFPPNFVHSLDGSHMMMTAVACRKAGICFAGVHDSYWTHACDVDELNRTLREK

SoRpoTmp MVRRQKSAFPPNFVHSLDGSHMMMTAVACKHAGLNFAGVHDSYWTHACDVDQMSSILREK

AtRpoTm MARRQMTAFAPNFIHSLDGSHMMMTAVACNRAGLSFAGVHDSFWTHACDVDVMNTILREK

BoRpoTm MARKQMTAFAPNFVHSLDGSHMMMTAVACNKAGLSFAGVHDSFWTHACDVELMNNILREK

NaRpoTp LVKRQKTAFPPNFVHSLDGSHMMMTAVACKLAGLNFAGVHDSYWTHACDVDDMSRILRLK

PpRpoTmp1 LVQRQKSAFPPNFVHSLDSTHMMMTALACQEAGLTFAGVHDSYWTHAGDVEQMNSLLREK

PpRpoTmp2 LASRQRSAFPPNFVHSLDSSHMMMTALACSKAGLTFAGVHDSYWTHAGDVENMNVILRKN

PpRpoTm LKTRQRSAFAPNFIHSLDSTHMMLTALASNQAGISFAGVHDSFWTHAGDVDVLNKLTREK

SmRpoTm LAARQRSAFPPNFVHSMDGSHMMMTAVACQEAGLTFAGVHDSFWTHAGDVERMNVILREK

MspecRpoT AKAKQRSAFPPNFIHSIDSAHMMKTALACHEAGLTFAGVHDSFWTHAGDVPEMSRHIREK

MipuRpoT QKQKQKSAFPPNFIHSVDSAHMMKTALACQRRDIAFAGVHDSFWTHARDVDAMNEVIRDT

OlRpoT MKVKQRSAFPPNYIHSIDSSHMMRTAIACVDAGLTFAGVHDSFWTHATDVDTMNVILREK

OtRpoT MKVKQRSAFPPNYIHSIDSSHMMKTAVACVDAGLTFAGVHDSFWTHACDIDTMNKILREK

PotRpoTp1 FVELYNMPILENLLEDFQTSYPTLQ--FPPLPERGNFDLQKVLRSPYFFN

PotRpoTp2 FVELYNMPILENLLEDFQTSYPTLK--FPSLPERGNFDLQEVLRSPYFFN

VvRpoTp FVELYSMPILENLLESFQTSHPTLT--FPPLPDRGNFDLREVLESPYFFN

NsRpoTp FVELYSMPILEDLLESFQNSYPALT--FPPLPKRGDFDLVEVLESPYFFN

AtRpoTp FVELYNTPILEDLLQSFQESYPNLV--FPPVPKRGDFDLKEVLKSQYFFN

SoRpoTp FVELYNMPILENLLESFQTAYPAST--FPPLPERGDFDLEEVLESPYFFN

ZmRpoTp FVELYSMPILENLLEEFQTSFPTLE--FPPCPPQGNFDVREVLTSTYFFN

SbRpoTp FVELYSMPILDNLLEEFQMAFPTLE--FPPCPPQGNFDVREVLTSTYFFN

OsRpoTp FVELYSMPILENLLKEFQTSFPTLE--FPPCPSQGDFDVREVLASTYFFN

HvRpoTp FVELYSMPILENLLEEFQTLFPTVE--FPPCPAQGNFDVREVLTSTYFFN

TaRpoTp FVELYSMPILENLLEEFQTLFPTVE--FPPCPAQGNFDVREVLTSTYFFN

SbRpoT1 FVELYDTPILENLLESFEKSFPKLK--FPPLPERGDFDMKEVLESPYFFN

ZmRpoTm FVELYDTPILENLLESFEKSFPKLK--FPPLPERGDFDMKEVLESTYFFN

HvRpoTm FVELYDAPILENLLESFETSFPKLK--FPPLPERGNFDMKDVLQSTYFFN

TaRpoTm FVELYDAPILENLLESFETSFPKLK--FPPLPERGNFDMKDVLQSTYFFN

OsRpoTm FVELYDTPILENLLESFEKSFPELK--FPPLPERGDFDLTDVLGSPYFFN

NaRpoTm2 FVELYEQPILENLLESYQKSFPKLS--FPPLPDRGDFDLKEVLKSPYFFQ

NaRpoTm1 FVELYEQPILENLLEGFQKSFPKFS--FPPLPDRGDFDLKEVLQSTYFFN

VvRpoTm FVELYGMPILENLLEGFQKSFPTLN--FPPLPERGDFDLREVLESPYFFN

NsRpoTm FVELYDAPILENLLESFQQSFPDLQ--FPPLPERGDFDLREVLESPYFFN

PotRpoTm1 FVELYEAPILENLLESFQSSFPNLK--FPPLPERGVFDLKDVLRSTYFFN

PotRpoTm2 FVELYETPILENLLESFQSSFPNLK--FPPLPERGDFDLRDVLQSTYFFN

RcRpoTm FVELYEAPILENLLEGFQKSFPKLK--FPPLPDRGDFDLRDVLESPYFFN

CaRpoTm FVELYEQPILENLLESFQESFPTLQ--FPPLPERGDFDLREVLESPYFFN

NsRpoTmp FVELYEAPILEKLLESFQTSYPTLL--FPPLPERGDFDMRDVLESPYFFN

VvRpoTmp FVQLYETPILENLLESFQQSFPALE--FPPLPERGDFDLREVLESPYFFN

PotRpoTmp FVELYETPILKNLLESFQKSFPTLS--FPPLPEWGDFELRQVLESPYFFN

AtRpoTmp FVELYSQPILENLLESFEQSFPHLD--FPPLPERGDLDLKVVLDSPYFFN

CsRpoTmp FVELYDKPILEDLLESFEKSFPHLS--FPPLPERGDFDLREVLNSPYFFN

SoRpoTmp FVELYDQPILENLLETFQQSYPTLS--FPPLPDRGDFELRDVLDSPYFFN

AtRpoTm FVELYEKPILENLLESFQKSFPDIS--FPPLPERGDFDLRKVLESTYFFN

BoRpoTm FVELYDKPILENLLESFQKSFPGLT--FPPLPERGDFDLREVIRSPYFFN

NaRpoTp FVELYSMPILENLLESFQTSFPTLV--FPPLPDRGDFDLQEVLESPYFFN

PpRpoTmp1 FVELYSQPVLENLLKSFQERFPTLV--FPEVPARGDLDLKEVLRAPYFFN

PpRpoTmp2 FVKLYKQPILENLLLDFQTQFPDLV--FPEVPARGDLDLKEVLKSPYFFN

PpRpoTm FVELYSYPILENLLLGFQRRYADLT--FPPVPERGVLDIREVLKAPYFFN

SmRpoTm FVELYEQPILENLLESFQKRWPKLK--FPDLPVRGDLDLKEVLSSPYFFN

MspecRpoT FIELHSEPLLEMLYEELKEKYPEVAHEIPPPPPMGNMDIEQVKDSIYFFS

MipuRpoT FVELHSEPLMTTLYEELRETYPEAADEIPKPPPLGELDLNEVKKSRYFFS

OlRpoT FIEVHKEPLLENLYHEFRANYPDVADEFPQPPAPGDLDLDVVQDSVYFFS

OtRpoT FIEVHSEPLLENLYEELRESYPDVADKFPRPPKPGDLDLNVVRDSVYFFS

Sequences and their accession numbers:

AtRpoTm - *Arabidopsis thaliana* (P92969); AtRpoTp - *A. thaliana* (O24600); AtRpoTmp - *A. thaliana* (CAC17120); BoRpoTm - *Brassica oleracea* var. *alboglabra* (XP_002308414.1), CaRpoTm - *Chenopodium album* (CAA69305); CsRpoTmp - *Cleome spinosa* (DQ415921); HvRpoTm - *Hordeum vulgare* (AJ586899); HvRpoTp - *H. vulgare* (AJ507396); MipuRpoT - *Micromonas pusilla* (EEH56417.1); MspecRpoT - *Micromonas sp*. RCC299 (XP_002503703.1); NaRpoTm1 - *Nuphar advena* (FN811768), NaRpoTm2 - *N. advena* (FN820498), NaRpoTp - *N. advena* (FN811769); NsRpoTm - *Nicotiana sylvestris* (AJ416568); NsRpoTp - *N. sylvestris* (AJ302020); NsRpoTmp - *N. sylvestris* (AJ302019); OlRpoT - *Ostreococcus lucimarinus* (database entry not yet available, sequence derived from the corresponding genome raw data and EST data; U. Richter, unpublished data); OsRpoTm - *Oryza sativa* (AB096014); OsRpoTp - *O.sativa* (AB096015); OtRpoT - *Ostreococcus tauri* (CAL55557.1); PotRpoTm1, PotRpoTm2, PotRpoTmp, PotRpoTp1, PotRpoTp2 - *Populus trichocarpa*, sequences derived from five RpoT polymerase genes identified in the genome of poplar (see http://www.phytozome.net/poplar; U. Richter, unpublished data); PpRpoTmp1 -  *Physcomitrella patens* (CAC95163); PpRpoTmp2 - *P. patens* (CAC95164); PpRpoTm - *P. patens* (see Legend to Figure 5); RcRpoTm - *Ricinus communis* (database entry not yet available, sequence derived from the corresponding genome raw data and EST data; U. Richter, unpublished data); SbRpoT1m - *Sorghum bicolor* (XM_002460990), SbRpoTp - *S. bicolor* (XM_002437329); SmRpoT -  *Selaginella moellendorfii* (CAP70041); SoRpoTp - *Spinacia oleracea* (Y18853); SoRpoTmp - *S. oleracea* (Y18852); TaRpoTm - *Triticum aestivum* (AAF32492); TaRpoTp - T*. aestivum* (AAB01085); VvRpoTm - *Vitis vinifera* (AM483136), VvRpoTp - *V. vinifera* (AM453066), VvRpoTmp - *V. vinifera* (AM488491); ZmRpoTm - *Zea mays* (AAD22977); ZmRpoTp - *Z. mays* (AAD22976).
